# The pluripotent regulatory circuitry connecting promoters to their long-range interacting elements

**DOI:** 10.1101/gr.185272.114

**Published:** 2015-04

**Authors:** Stefan Schoenfelder, Mayra Furlan-Magaril, Borbala Mifsud, Filipe Tavares-Cadete, Robert Sugar, Biola-Maria Javierre, Takashi Nagano, Yulia Katsman, Moorthy Sakthidevi, Steven W. Wingett, Emilia Dimitrova, Andrew Dimond, Lucas B. Edelman, Sarah Elderkin, Kristina Tabbada, Elodie Darbo, Simon Andrews, Bram Herman, Andy Higgs, Emily LeProust, Cameron S. Osborne, Jennifer A. Mitchell, Nicholas M. Luscombe, Peter Fraser

**Affiliations:** 1 Nuclear Dynamics Programme, The Babraham Institute, Babraham Research Campus, Cambridge CB22 3AT, United Kingdom;; 2 University College London, UCL Genetics Institute, Department of Genetics, Evolution and Environment, University College London, London WC1E 6BT, United Kingdom;; 3 Cancer Research UK London Research Institute, London WC2A 3LY, United Kingdom;; 4 EMBL European Bioinformatics Institute, Wellcome Trust Genome Campus, Hinxton, Cambridge CB10 1SD, United Kingdom;; 5 Department of Cell and Systems Biology, University of Toronto, Toronto, Ontario M5S 3G5, Canada;; 6 Bioinformatics Group, The Babraham Institute, Babraham Research Campus, Cambridge CB22 3AT, United Kingdom;; 7 Agilent Technologies, Inc., Santa Clara, California 95051, USA;; 8 Okinawa Institute for Science and Technology Graduate University, 1919-1 Tancha, Onna-son, Kunigami-gun, Okinawa 904-0495, Japan

## Abstract

The mammalian genome harbors up to one million regulatory elements often located at great distances from their target genes. Long-range elements control genes through physical contact with promoters and can be recognized by the presence of specific histone modifications and transcription factor binding. Linking regulatory elements to specific promoters genome-wide is currently impeded by the limited resolution of high-throughput chromatin interaction assays. Here we apply a sequence capture approach to enrich Hi-C libraries for >22,000 annotated mouse promoters to identify statistically significant, long-range interactions at restriction fragment resolution, assigning long-range interacting elements to their target genes genome-wide in embryonic stem cells and fetal liver cells. The distal sites contacting active genes are enriched in active histone modifications and transcription factor occupancy, whereas inactive genes contact distal sites with repressive histone marks, demonstrating the regulatory potential of the distal elements identified. Furthermore, we find that coregulated genes cluster nonrandomly in spatial interaction networks correlated with their biological function and expression level. Interestingly, we find the strongest gene clustering in ES cells between transcription factor genes that control key developmental processes in embryogenesis. The results provide the first genome-wide catalog linking gene promoters to their long-range interacting elements and highlight the complex spatial regulatory circuitry controlling mammalian gene expression.

Mammalian development and cell identity critically depend on the function of regulatory DNA elements (such as enhancers, silencers, and insulators) to establish spatiotemporal gene expression programs. Although recent advances in next generation sequencing have enabled the large-scale identification of regulatory DNA elements in mammalian genomes, which genes they regulate remains largely unknown. Distant genomic regions can be brought into close spatial proximity through specific chromosomal interactions that play a key role in gene expression control (Bulger and Groudine 2011). For example, developmental enhancers can be located at considerable genomic distances from the gene promoters they regulate, often bypassing several promoters located in the intervening DNA sequence to interact with their target genes (Carvajal et al. 2001; Carter et al. 2002; Spitz et al. 2003; Sagai et al. 2005; Jeong et al. 2006; Pennacchio et al. 2006; Ruf et al. 2011; Marinic et al. 2013). These findings challenge the concept of inferring regulatory interactions from genomic proximity, which underlies the widely used strategy to assign enhancers to the nearest gene promoter. An alternative strategy is to link promoters with enhancers based on capturing their physical contacts, because direct interactions between enhancers and promoters are central to the dominant models for enhancer function (Bulger and Groudine 2011). In strong support of these models, experimental tethering between an enhancer and its target gene can induce gene transcription even in the absence of a key transcriptional activator (Deng et al. 2012). A major task toward unraveling gene expression circuitry is to link, on a genome-wide scale, regulatory sequences to the gene promoters they control.

Preferential chromosomal organization is not confined to contacts between genes and regulatory elements. Intra- and interchromosomal associations between genes have been detected in a range of nuclear processes, including gene activation (Osborne et al. 2004, 2007; Spilianakis et al. 2005; Apostolou and Thanos 2008), gene silencing (Bantignies et al. 2011; Engreitz et al. 2013), and recombination (Skok et al. 2007; Zhang et al. 2012). Spatial coassociations have also been observed between coregulated genes (Schoenfelder et al. 2010; Apostolou et al. 2013; de Wit et al. 2013; Denholtz et al. 2013). These findings suggest that spatial proximity between specific genomic elements, in addition to shaping 3D genome architecture, may influence genome function (Fanucchi et al. 2013).

The 3C technique (Dekker et al. 2002) and its derivatives have revolutionized the study of 3D genome organization by providing the means to capture spatial proximity between genomic regions. Variations of 3C have focused on interactions for a small number of genomic bait regions (4C) (Simonis et al. 2006; Zhao et al. 2006; van de Werken et al. 2012), interactions within specific genomic domains (5C) (Dostie et al. 2006; Sanyal et al. 2012), or involving a particular protein of interest (ChIA-PET) (Fullwood et al. 2009; Handoko et al. 2011; Zhang et al. 2013). Hi-C, a genome-wide adaptation of 3C (Lieberman-Aiden et al. 2009), has the potential to capture the ensemble of chromosomal interactions within a cell population. However, the vast complexity of mammalian 3C or Hi-C libraries (estimated to contain up to 10^11^ unique pair-wise interactions) (Belton et al. 2012) impedes their analysis at a resolution required to identify interactions between specific elements, such as promoters and enhancers. To overcome this limitation, we and others have incorporated a sequence capture step to enrich 3C (Hughes et al. 2014) or Hi-C (Dryden et al. 2014) libraries for chromosomal interactions involving a few hundred specific bait regions. These studies demonstrate the feasibility of capturing specific interactions in 3C/Hi-C libraries, but a genome-wide approach enabling the systematic, unbiased, and high-resolution interrogation of chromosomal interactions for tens of thousands of genomic elements simultaneously, independent of their activity or bound proteins, is currently lacking.

Here we combine Hi-C with sequence capture enrichment (CHi-C for Capture Hi-C) for 22,225 annotated gene promoters in the mouse genome. We apply promoter CHi-C to mouse embryonic stem cells (ESCs) and mouse fetal liver cells (FLCs), creating the first genome-wide map of interaction profiles for all annotated mouse gene promoters in pluripotent and committed/differentiated cells.

## Results

### Promoter capture Hi-C

**Figure 1.**
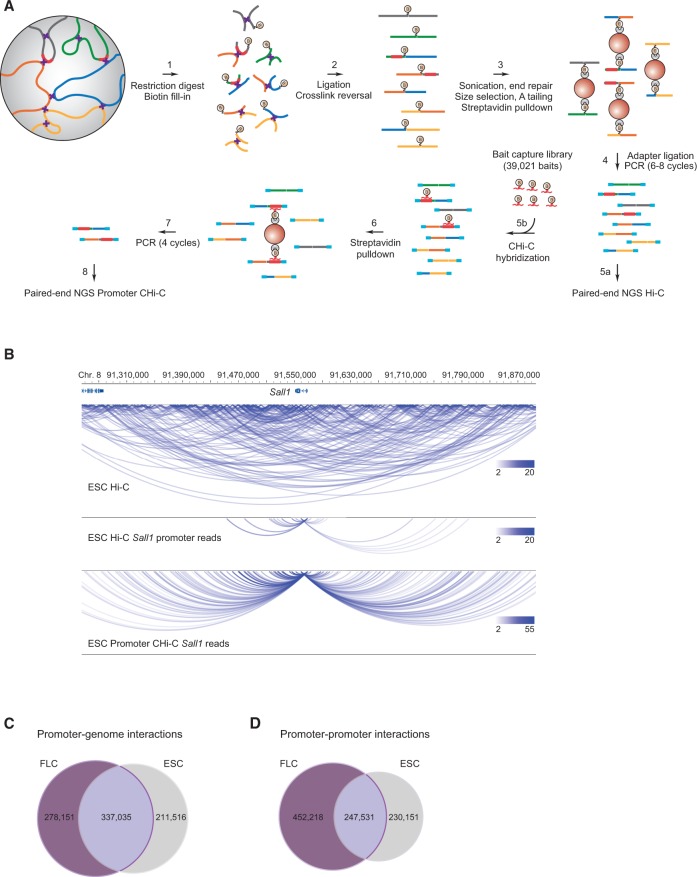
Promoter capture Hi-C. (*A*) Experimental strategy: Hi-C libraries were either directly interrogated by massively parallel paired-end sequencing (step 5a) or subjected to promoter CHi-C (steps 5b–8). For promoter CHi-C, the Hi-C library is hybridized to the RNA capture library (“bait”) in solution, followed by streptavidin pulldown of Hi-C library ligation products containing promoters targeted by the biotin-RNA baits (22,225 promoters in the mouse genome). The resulting promoter CHi-C library is analyzed by massively parallel paired-end sequencing. Chromosomal regions are depicted in blue, green, gray, orange, and yellow; promoters are depicted in red; and sequencing adapters in light blue. Biotin moieties are symbolized by an encircled “B,” and formaldehyde crosslinks are represented by purple crosses. RNA bait molecules are represented by red fragments connected to a biotin moiety. (*B*) The chromosomal interactome of the *Sall1* locus in ESCs. Shown are unfiltered read pairs from Hi-C data for a 0.6-Mb region containing the *Sall1* gene (*top*), *Sall1* promoter-contacting read pairs from the same Hi-C data (*middle*), and *Sall1* promoter-contacting read pairs from promoter CHi-C (*lower*). Hi-C and CHi-C data sets were adjusted to the same number of overall sequence reads. Interactions are displayed using the WashU EpiGenome Browser (Zhou et al. 2013). (*C*) Unique and shared promoter–genome significant interactions after GOTHiC filtering in ESCs and FLCs. (*D*) Unique and shared promoter–promoter significant interactions after GOTHiC filtering in ESCs and FLCs.

To generate chromosomal interaction maps for all annotated promoters in the mouse genome, highly complex Hi-C libraries were subjected to solution hybrid capture with a custom-designed collection of 39,021 biotinylated RNA “baits” targeting 22,225 annotated promoter-containing restriction fragments (Fig. 1A; Supplemental Table 1). We generated two promoter CHi-C biological replicates for both ESCs and FLCs and sequenced them to high depth. In total, we analyzed more than 1.9 billion CHi-C paired-end sequence reads (ditags), which were reduced to 754 million uniquely mapped ditags after data filtering (HiCUP Hi-C analysis pipeline) (see Methods). Promoter bait coverage was highly correlated between the two biological promoter CHi-C replicates (Spearman correlation *r* = 0.91 for ESC and *r* = 0.95 for FLC). Sequence capture efficiencies, defined as the percentage of ditags with at least one end mapping to a targeted promoter, were 71.1% for ESC and 65.6% for FLC, in line with previously reported sequence capture approaches (Gnirke et al. 2009). We removed off-target and exact sequence duplicate read pairs from our data (Supplemental Table 2), since control barcoding experiments demonstrated that exact duplicates arise from preferential PCR amplification rather than independent ligation events (SW Wingett, S Schoenfelder, M Furlan-Magaril, T Nagano, P Fraser, S Andrews, in prep.). Compared to our precapture Hi-C libraries, and to previously published Hi-C libraries (Dixon et al. 2012), promoter CHi-C resulted in >10-fold enrichment of read-pairs involving promoter elements (Fig. 1B; Supplemental Fig. 1A,B).

Capturing promoter fragments markedly enriches their interacting fragments, thus reducing the overall library complexity compared to a corresponding precapture Hi-C library. In order to obtain an equivalent number of promoter reads, a Hi-C library would need to be sequenced up to 19-fold greater depth. With this increased power, promoter CHi-C enables the identification of statistically significant promoter interactions at the restriction-fragment level. To this end, we developed an interaction-calling algorithm called GOTHiC (Genome Organisation Through Hi-C) (B Mifsud, I Martincorena, E Darbo, R Sugar, S Schoenfelder, P Fraser, NM Luscombe, in prep.). GOTHiC accounts for biases in Hi-C experiments by considering that these will be represented by the total coverage of the interacting fragments. Using the fragment coverage, GOTHiC uses a cumulative binomial test to calculate the probability of having two fragments linked by the observed number of reads. *P*-values are corrected for multiple testing with the Benjamini-Hochberg procedure (Benjamini and Hochberg 1995), and significant interactions are called using an FDR < 0.05. We focused on promoter interactions that were present in both replicates and further filtered the significant interaction set by interaction strength (see Methods). Promoter CHi-C results in two types of paired-end sequence reads: read-pairs in which one end maps to a promoter fragment and the other maps to a nonpromoter fragment (promoter–genome contacts) (Fig. 1C); and read-pairs in which both ends map to promoter fragments (promoter–promoter contacts) (Fig. 1D). Because these two classes of ditags potentially represent different types of interactions, we analyzed them separately.

**Figure 2.**
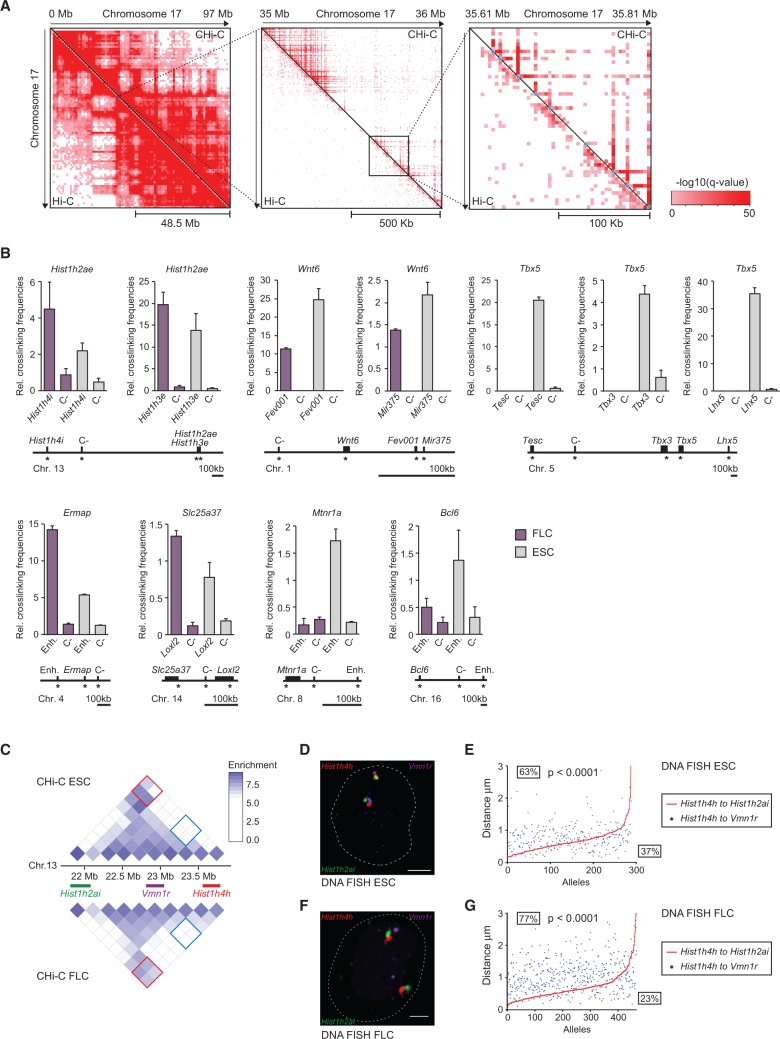
Validation of promoter interactions. (*A*) Hi-C and promoter CHi-C contact maps after GOTHiC filtering for significant interactions: whole chromosome view of mouse Chromosome 17 (*left*), and 1-Mb (*middle*) and 200-kb subregions (*right*) encompassing the *Pou5f1* gene locus. Individual promoter bait restriction fragments are marked by light blue dots in the *right* panel. Color intensity corresponds to the significance of the interaction, −log_10_(*q*-value) from GOTHiC. (*B*) Validation of CHi-C results by 3C-qPCR. Graphs showing the relative crosslinking frequencies of promoter restriction fragments (*top*) with another promoter, putative enhancer (Enh) or control, noninteracting fragments (C-), as depicted in the graphs and the maps *below*. Interactions identified by promoter CHi-C present in both cell types (*Hist1h2ae*), preferential in ESCs (*Wnt6*, *Tbx5, Mtnr1a, Bcl6*), or preferential in FLCs (*Ermap, Slc25a37*) are shown. Control fragments (C-) were identified as noninteracting, or interacting at lower frequencies by CHi-C, compared to the interacting fragments in the respective cell type. Asterisks denote the position of the primers used in 3C-qPCR. (*C*–*G*) Validation of CHi-C results by triple-label 3D DNA FISH. (*C*) Promoter CHi-C contact maps for a ∼2-Mb region on mouse Chromosome 13 in ESCs (*top*) and FLCs (*below*), encompassing the *Hist1h2ai*, *Vmn1r*, and *Hist1h4h* loci as shown. Contact enrichment between *Hist1h4h* and *Vmn1r* loci are marked by blue squares on the contact maps, and contact enrichment between *Hist1h4h* and *Hist1h2ai* are marked by red squares. (*D*) and (*F*) Representative triple-label 3D DNA FISH in ESCs (*D*) and FLCs (*F*), DNA FISH signals for the *Hist1h2ai* locus (green), the *Vmn1r* locus (purple), and the *Hist1h4h* locus (red). Scale bar, 2 μm. (*E*) and (*G*) Interprobe distance measurements of triple-label 3D DNA FISH in ESCs (*E*) and FLCs (*G*). Shown are the ranked interprobe distances between *Hist1h4h* and *Hist1h2ai* (red line) with the corresponding interprobe distance between *Hist1h4h* and *Vmn1r* (blue dots) per allele. Percentages *above* the red line indicate the frequency at which the distance between *Vmn1r* and *Hist1h4h* is greater than the distance between *Hist1h2ai* and *Hist1h4h*, whereas percentages *below* the line indicate the frequency at which the distance between *Vmn1r* and *Hist1h4h* is less than the distance between *Hist1h2ai* and *Hist1h4h. P*-values: χ^2^ test comparing the distance distributions between *Vmn1r* and *Hist1h4h* to the distance between *Hist1h2ai* and *Hist1h4h*.

GOTHiC detected 317,271 genomic fragments engaged in 548,551 significant, reproducible interactions with 21,748 promoters in ESCs. In FLCs, we detected 311,475 genomic fragments involved in 615,186 significant, reproducible long-range interactions with 21,431 promoters (Fig. 1C). In both cell types, >99.9% of the significant promoter–genome contacts were between promoters and elements located on the same chromosome. The majority of promoter–genome interactions (59%) were unique to either ESCs or FLCs, indicating strong tissue-specific promoter interactomes. GOTHiC also detected 477,682 and 699,749 significant promoter–promoter contacts in ESCs and FLCs, respectively (Fig. 1D). More than 73% of these contacts were unique to either ESCs or FLCs, demonstrating robust tissue-specific 3D genome organization of promoters. At the chromosomal level, Hi-C and CHi-C contact maps provide a similar coarse-grained view of 3D genome topology (Fig. 2A). However, in contrast to Hi-C, promoter CHi-C enables the identification of statistically significant long-range promoter interactions at the restriction fragment level (Fig. 2A).

To validate promoter capture Hi-C, we compared our data sets to published 3C and 4C data. ESC-specific long-range interactions involving the *Phc1* (Kagey et al. 2010) and *Nanog* (Levasseur et al. 2008) genes were recapitulated in our data (Supplemental Fig. 1C,D), as was an interaction between *Pou5f1* and a putative enhancer element (Supplemental Fig. 1E; van de Werken et al. 2012). Similarly, known erythroid cell-specific enhancer–promoter interactions in the *Hbb* (Mitchell and Fraser 2008) and *Hba* (Vernimmen et al. 2007) gene loci were accurately detected in FLCs (Supplemental Fig. 1F,G).

The promoter-interacting regions identified by promoter CHi-C include regulatory elements that are required for appropriate expression of their target genes, such as enhancers controlling *Hba* (Supplemental Fig. 1F; Anguita et al. 2002), *Hbb* (Supplemental Fig. 1G; Bender et al. 2001, 2012), *Sox2* (Zhou et al. 2014; data not shown), and *Tal1* transcription (Supplemental Fig. 1H; Ferreira et al. 2013). These examples indicate that CHi-C uncovers functional chromosomal interactions and illustrate the potential of promoter CHi-C to link gene promoters to the regulatory elements controlling their expression.

We further validated a subset of shared and tissue-specific promoter–genome and promoter–promoter interactions using quantitative 3C (3C-qPCR). In all cases tested, we detected higher 3C interaction frequencies for contacting genomic fragments identified by promoter CHi-C in the appropriate tissue than for more proximally located noninteracting regions (Fig. 2B). Finally, to validate promoter CHi-C by an independent method, we assessed selected long-range contacts by triple-label 3D DNA FISH (Supplemental Fig. 2; Fig. 2C–G). The results show that contacting regions separated by multiple megabases are more frequently in close spatial proximity than intervening control regions in the appropriate cell types. Collectively, the comparison to published data and validation by 3C and 3D DNA FISH demonstrate that promoter CHi-C accurately identifies promoter-interacting, long-range chromosomal elements and multiscale, tissue-specific genome architecture.

### Promoter–genome interactomes

**Figure 3.**
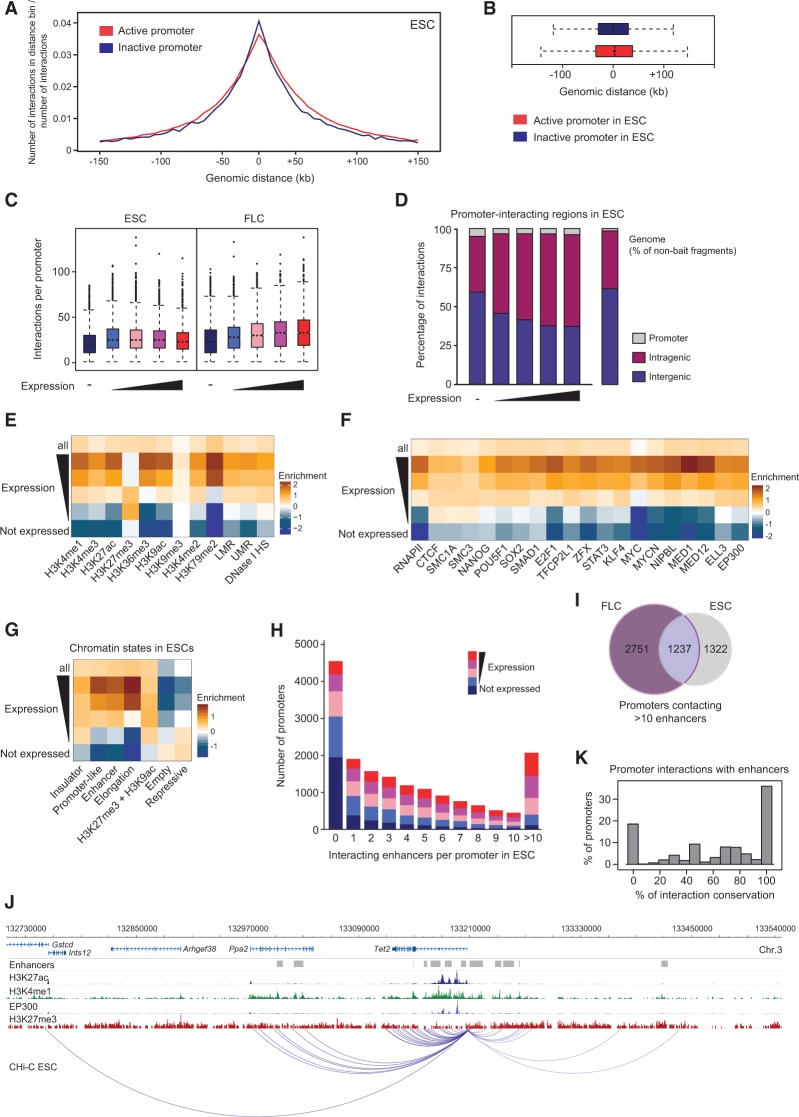
Hallmarks of promoter-interacting regions. (*A*) Composite profile showing the proportion of promoter–genome interactions for 5-kb distance bins upstream of and downstream from the transcription start sites for active (red) and inactive (blue) promoters in ESCs. (*B*) Genomic range of interactions for active (red) and inactive (blue) promoters in ESCs. (*C*) Number of promoter–genome interactions in ESCs and FLCs, separated by expression categories. (*D*) Intra- and intergenic distribution of promoter-interacting regions in ESCs, with genes driven by the promoters separated in expression categories (HindIII fragments encompassing exonic or intronic sequences are classed as “intragenic” here). The distribution of intragenic and intergenic sequences in the mouse genome is shown on the *right*. (*E*–*G*) Heat maps showing the enrichment/depletion for histone modifications (*E*), chromatin proteins (*F*), and chromatin states (*G*) in promoter-interacting regions in ESCs, for all promoters and separated by expression of the interacting promoters, compared to nonbait regions. (UMR) Unmethylated region; (LMR) low-methylated region (Stadler et al. 2011). (*H*) Number of promoters from each expression category interacting with between zero and more than 10 genomic elements with the hallmarks of enhancers in ESCs. (*I*) Unique and overlapping promoters interacting with multiple (>10) enhancer-like elements in ESCs and FLCs. (*J*) Example of a promoter (driving the *Tet2* gene) contacting multiple enhancers in ESCs. (*K*) Conservation of promoter–enhancer contacts between ESCs and FLCs. Shown is the percentage of promoters that share 0%, 10%, 20%, etc., of their interactions with enhancer-like elements. Only enhancers active in both cell types (ESC and FLC) have been included in the analyses.

To obtain a generalized view of the genomic range of promoter interactions, we plotted the average promoter interaction frequency against increasing genomic distance from the promoters (Fig. 3A,B; Supplemental Fig. 3A,B). These profiles confirm the inverse relationship between genomic distance and interaction frequency that has previously been reported in 4C, 5C, and Hi-C data sets (Gibcus and Dekker 2013). We found that active promoters undergo significantly fewer short-range and more long-range interactions than inactive promoters (*P*-value < 2.2 × 10^−16^; Ansari-test), suggesting that the activity of a gene promoter is linked to the range of its chromosomal interactome (Fig. 3A,B; Supplemental Fig. 3A,B). Increasing gene expression is positively correlated with the number of promoter interactions in FLCs, but less so in ESCs (Spearman correlation) (Fig. 3C); whereas the average number of interactions per promoter is comparable in both cell types (Supplemental Fig. 3C). Promoter-interacting fragments in both cell types show a higher sequence conservation compared to all nonbait fragments (*P*-value < 2 × 10^−16^) (Supplemental Fig. 3D). We found that interactions between promoters and intragenic sequences are more prevalent than interactions with intergenic regions and that this preference increases with promoter expression level (Spearman correlation) (Fig. 3D; Supplemental Fig. 3E). This may reflect the fact that regulatory elements can be located within genes they control, or indeed within neighboring or distal genes.

### Epigenetic modifications at distal interacting sites

To assess the regulatory potential of promoter–genome interacting sites, we integrated our promoter CHi-C data with published epigenome data sets. In total, we examined data for 10 different histone modifications, DNase I hypersensitivity, and low and unmethylated DNA regions (Supplemental Table 3). We found high levels of enrichment of active histone marks (H3K4me1, H3K4me2, H3K4me3, H3K9ac, H3K27ac, H3K36me3) at distal promoter-interacting sites that correlated with promoter expression level in ESCs and FLCs (Fig. 3E; Supplemental Fig. 3F). Promoters of highly expressed genes interact with regions that are highly enriched for “active” histone marks, whereas regions interacting with moderately expressed genes show a less pronounced enrichment. Promoters of weakly expressed and silent genes interact with regions that are depleted for active histone marks (Fig. 3E). In contrast, the repressive histone mark H3K27me3 is enriched at regions interacting with promoters of poorly expressed genes (Fig. 3E; Supplemental Fig. 3F). These results suggest that the promoter-interacting sites identified show marks of regulatory potential appropriate to the activity level of the genes they contact.

### *Trans*-acting factor occupancy in promoter-interacting regions

We further characterized promoter-interacting regions in ESCs and FLCs by assessing *trans-*acting factor occupancy (Supplemental Table 3). In total, almost 43% (135,944/317,271) of the promoter-interacting fragments uncovered by promoter CHi-C in ESCs harbor at least one of the chromatin marks analyzed, indicative of potential biological function (Supplemental Table 4). In general, we found high levels of enrichment of various transcriptional regulatory factors occupying regions interacting with moderately to highly expressed gene promoters (Fig. 3F; Supplemental Fig. 3G). The same trend is seen in ESCs for the Mediator complex (NIPBL, MED1, MED12), which has been implicated in the establishment and/or maintenance of chromosomal interactions (Kagey et al. 2010; Phillips-Cremins et al. 2013). Binding sites for EP300 and ELL3, two chromatin proteins that mark enhancer sequences (Chen et al. 2008; Visel et al. 2009; Lin et al. 2013), are also enriched in genomic regions interacting with expressed gene promoters (Fig. 3F). Genomic regions interacting with nonexpressed gene promoters are either not enriched or depleted for occupancy by these factors. As expected, none of the analyzed chromatin proteins were enriched in promoter-interacting regions when randomized ChIP-seq data sets were used (data not shown).

To gain further insight into the features of promoter-interacting regions, we defined a set of chromatin states in ESCs, characterized by distinct combinations of factor occupancy and histone modifications (Supplemental Fig. 3H), and analyzed their enrichment in promoter-interacting regions (Fig. 3G). The chromatin state results corroborate those obtained with individual ChIP-seq data sets (Fig. 3E,F), and show that promoters of expressed genes preferentially interact with chromatin associated with transcriptional activation (“Enhancer,” “Promoter-like,” “Elongation”). Conversely, inactive gene promoters preferentially associate with repressive chromatin (Fig. 3G). Collectively, these results suggest that the long-range promoter-interacting elements identified have strong regulatory potential.

### Enhancer–promoter contacts

Previous studies have identified transcriptional enhancers in ESCs and FLCs based on specific combinations of chromatin marks, such as H3K4me1, H3K27ac, and EP300 binding (Shen et al. 2012). Our results assign more than two-thirds of all such identified enhancers in the cell types analyzed (67.6% in ESCs; 70.3% in FLCs) to potential target genes. The remaining predicted enhancers may act via mechanisms that do not involve direct promoter contact, interact too transiently with their target genes to be captured, or interact in response to specific signals. Our data also show that only about one in five promoter-interacting elements identified (17.7% for ESCs; 19.7% for FLCs) are predicted enhancers (Shen et al. 2012), suggesting that other types of elements may contribute to promoter regulation.

Previous studies have suggested that mammalian genomes harbor more than one million enhancers, far outnumbering gene promoters (The ENCODE Project Consortium 2012; Shen et al. 2012; Calo and Wysocka 2013). The extent to which multiple enhancers interact with the same target gene, and whether specific enhancers drive expression of the same target gene in different cell types, is largely unknown. We found that 26.6% of all promoters analyzed do not interact with any putative enhancer elements in ESCs (Fig. 3H); and as expected, inactive genes are overrepresented in this category. A total of 39.5% of promoters interact with several (2–10) enhancers, whereas 12.1% of promoters interact with multiple (more than 10) enhancers (Fig. 3H–J). Gene ontology analysis indicates that gene promoters interacting with more than 10 enhancers specifically in ESCs are enriched in developmental pathways, whereas genes driven by promoters interacting with more than 10 enhancers only in FLCs are enriched in metabolic functions (cumulative hypergeometric test with *P*-values corrected for multiple testing) (Supplemental Fig. 3I,J). In general, we observed a positive correlation between gene expression level and the number of interacting enhancer elements (Spearman correlation; *r* = 0.975 and *P* = 0.005 for both ESCs and FLCs), suggesting that additive effects of enhancers promote increased expression (Fig. 3H; Supplemental Fig. 3I). Less than half of the enhancers present in ESCs are also present in FLCs, and only a minority of these common enhancers are contacted by the same promoters in both cell types (Fig. 3K; Supplemental Fig. 3K). This finding indicates that extensive rewiring of enhancer–promoter contacts occurs during development.

### Highly connected enhancers and super-enhancers

**Figure 4.**
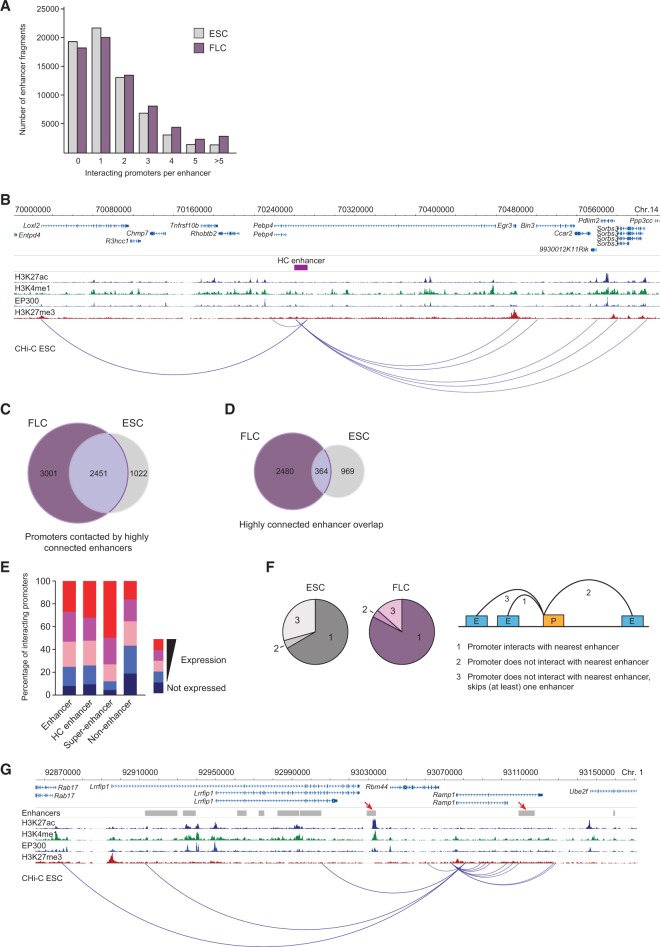
Promoter-enhancer 3D circuitry. (*A*) Number of enhancer elements interacting with between zero and more than five promoters in ESCs and FLCs. (*B*) Example of highly connected (HC) enhancer (represented by purple rectangle) contacting multiple gene promoters in ESCs. (*C*) Numbers of unique and overlapping promoters interacting with highly connected enhancers in ESCs and FLCs. (*D*) Numbers of unique and overlapping highly connected enhancers in ESCs and FLCs. (*E*) Percentage of promoters in the separate expression categories that contact enhancers, highly connected (HC) enhancers, super-enhancers, and nonenhancer elements in ESCs. (*F*) Percentages of active promoters interacting with the nearest enhancer (on the linear genomic map), a more distally located enhancer, or skipping (at least) one enhancer in ESCs and FLCs, as illustrated by schematic on the *right* ([P] promoter; [E] enhancer). (*G*) Example of an active promoter (*Ramp1*) bypassing proximal enhancers (red arrows) in ESCs.

We next asked whether enhancers are contacted by multiple promoters. The majority of enhancer-like elements are contacted by one to five promoters (69.1% in ESCs; 69.6% in FLCs), whereas a smaller fraction of highly connected enhancers (2% in ESCs; 4.1% in FLCs) are contacted by more than five promoters (Fig. 4A,B). The promoters contacting these highly connected (HC) enhancers show a high degree of overlap between ESCs and FLCs (Fig. 4C). However, the HC enhancers themselves are largely different between cell types (Fig. 4D; Supplemental Fig. 4A), suggesting that highly connected enhancers represent a class of tissue-specific hub enhancers that coordinate the expression of multiple genes expressed in both cell types. The expression levels of genes interacting with highly connected enhancers are similar to genes contacting other enhancer elements (Fig. 4E). These characteristics distinguish highly connected enhancers from super-enhancers, which differ from “regular” enhancers in both domain size and occupancy of chromatin proteins (Whyte et al. 2013). The 231 super-enhancers identified in murine ESCs are located in the genomic proximity of, and have been proposed to associate with, 210 key genes controlling cellular identity (Whyte et al. 2013). We found that 142 of these 210 genes interact with super-enhancers. In addition, we found 361 other genes that interact with super-enhancers, suggesting that super-enhancers control the expression of considerably more genes than previously appreciated (Supplemental Fig. 4B). Gene ontology analysis (cumulative hypergeometric test, *P*-values corrected for multiple testing) of this extended gene set indicates that super-enhancers contact key genes controlling cellular identity. Unlike highly connected enhancers, super-enhancers do not contact more promoters than other enhancer elements in the genome (Supplemental Fig. 4C), but highly expressed gene promoters are overrepresented among their targets (Fig. 4E). Interestingly, we found that nearly all genes that contact super-enhancers in ESCs (98.2%) also associate with other enhancers, suggesting that super-enhancers act in the context of larger 3D regulatory networks.

### Promoters frequently interact with distal enhancer elements

Enhancer–promoter interactions have been shown to bridge considerable genomic distances, looping out intervening DNA and often bypassing other promoters or enhancers that are located closer on the genomic map (Bulger and Groudine 2011). On a genome-wide level however, it is not known how frequently “enhancer skipping” occurs (i.e., how frequently a promoter skips over proximal enhancers for interactions with more distal enhancers). We found that in ESCs, 66.6% of active promoters interact with the nearest enhancer (Fig. 4F), whereas the remaining active ESC promoters interact with a more distal enhancer (4.1%) or bypass at least one enhancer (29.3%) (Fig. 4F,G). Enhancer skipping is also found in FLCs, albeit less prevalent than in ESCs (Fig. 4F; Supplemental Fig. 4D). If we consider individual enhancers and the location of promoters that contact them, we find that promoter skipping, from the viewpoint of enhancers, is also observed in ESCs and FLCs (Supplemental Fig. 4E). Our results demonstrate that enhancer–promoter contacts cannot be reliably inferred from genomic distance, consistent with chromosomal interaction data from human gene loci (Sanyal et al. 2012). Even in cases in which a promoter interacts with the nearest enhancer, 89.9% also interact with at least one more distal enhancer, indicating that the complexity of enhancer–promoter interactions is underestimated in the absence of spatial proximity data.

### Long-range interactions and 3D architectural features

**Figure 5.**
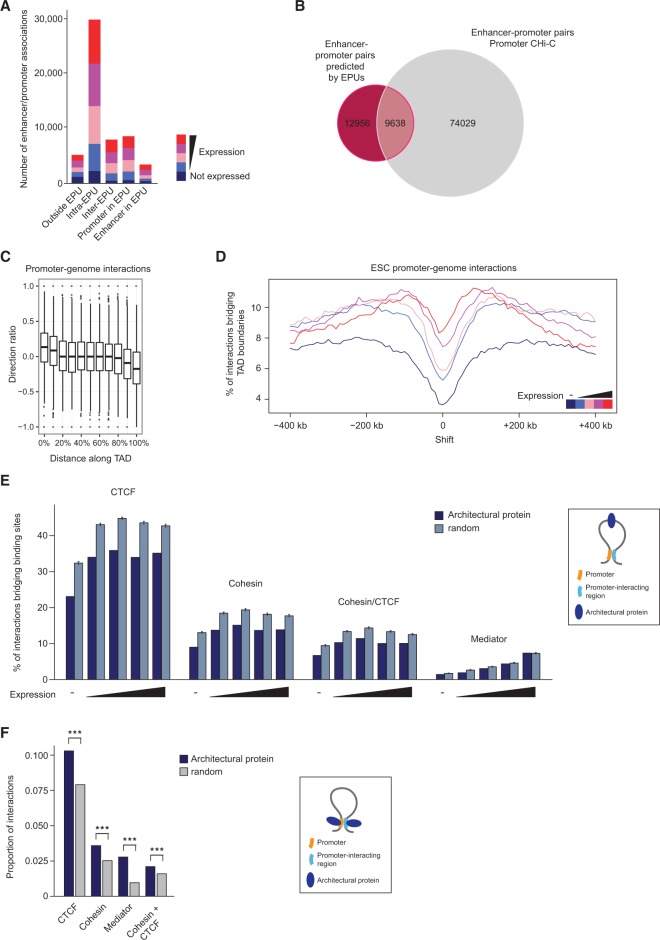
Demarcation of promoter interactions. (*A*) Number of interactions between promoters and enhancer elements with regard to the genomic position of enhancer promoter units (EPUs) (Shen et al. 2012) in ESCs, separated by expression categories. (*B*) Number of promoter–enhancer pairs predicted by EPUs (Shen et al. 2012) and promoter CHi-C. (*C*) Directionality of promoter–genome interactions in ESCs, relative to TAD boundaries. (*D*) Percentage of promoter–genome interactions bridging TAD boundaries, separated by expression categories. TAD boundary positions are set at zero and are then shifted artificially in 10-kb steps upstream and downstream. (*E*) Percentage of promoter interactions bridging binding sites of CTCF, cohesin (SMC1A), Mediator (MED12), and sites co-occupied by CTCF and cohesin in ESCs (as illustrated by schematic on the *right*) compared to randomized control sites. (*F*) Proportion of interactions in which the promoter and the interacting fragments are bound by the indicated proteins (CTCF, cohesin [SMC1A], Mediator [MED12], or CTCF and cohesin) in ESCs (as illustrated by schematic on the *right*), compared to randomized control sites.

Enhancer–promoter units (EPUs) have recently been defined based on the observation that coregulated enhancers and promoters form clusters on the linear genomic map (Shen et al. 2012). As expected, we found that the majority (54% in ESCs; 51.7% in FLCs) of enhancer–promoter interactions uncovered by CHi-C were between elements within the same EPUs (Fig. 5A; Supplemental Fig. 5A). However, we also identified a large number of interactions in which either the promoter (15.6%), the associating enhancer (6.2%), or both (9.4%) are located outside defined EPUs in ESCs (Fig. 5A; Supplemental Fig. 5A). In total, 42.7% of the 22,594 enhancer–promoter pairs predicted by EPUs in ESCs (Shen et al. 2012) were confirmed by our data (Fig. 5B). In addition, promoter CHi-C discovered 74,029 interactions between promoters and enhancer elements in ESCs that were not predicted by EPUs (Fig. 5B).

We next asked whether promoter interactions are limited by structural domains in eukaryotic genomes, such as topologically associating domains (TADs) (Dixon et al. 2012; Nora et al. 2012; Sexton et al. 2012) or lamina-associated domains (LADs) (Guelen et al. 2008). We found that only a minor fraction of promoter interactions occur within LADs (15.5%) or cross LAD boundaries (4.1%) (Supplemental Fig. 5B), consistent with the notion that LADs are gene-poor (Guelen et al. 2008; Peric-Hupkes et al. 2010). In contrast, our results show that most promoter–genome interactions occur within TADs, with only a minority bridging TAD boundaries (6% in ESCs; 9.1% in FLCs) (Supplemental Fig. 5C,D). We observed a marked directionality of promoter–genome interactions with regard to TAD boundaries (Fig. 5C). Notably, active promoters display a higher probability for inter-TAD interactions (χ^2^ test; ESC: X^2^ = 17131.9, *P*-value < 2.2 × 10^−16^; FLC: X^2^ = 19031.01, *P*-value < 2.2 × 10^−16^) (Supplemental Fig. 5C,D), which may reflect the fact that active genes in general engage in longer-range interactions compared to inactive genes (Fig. 3A,B; Supplemental Fig. 3A,B). This observation could also be explained by the fact that active promoters tend to be located close to TAD boundaries (Dixon et al. 2012). Nevertheless, we see clear local minima of interactions crossing TAD boundaries (Fig. 5D) even when only very long-range interactions (>500 kb) are considered (Supplemental Fig. 5E), consistent with the concept that TADs represent discrete regulatory domains.

We next looked for evidence that long-range interactions are hindered by sites bound by architectural proteins such as CTCF and cohesin (Phillips-Cremins et al. 2013). We found that the vast majority of CTCF binding sites genome-wide, including sites co-occupied by cohesin, are “bridged” by promoter–genome interactions (i.e., CTCF binding sites are located in the intervening sequence between promoters and the interacting genomic regions) (Supplemental Fig. 5F), supporting the idea that CTCF and CTCF/cohesin sites are not general blocks to long-range interactions. However, comparison to randomized controls suggest that CTCF and CTCF/cohesin sites are bridged by significantly fewer interactions than other genomic sites, even when CTCF/cohesin sites at TAD boundaries are removed from the analysis (*P*-values < 2.2 × 10^−16^) (Fig. 5E). This suggests that CTCF and CTCF/cohesin sites may selectively block long-range promoter interactions. We also found a significant number of promoter–genome interactions in which both fragments (the promoter and the interacting region) are bound by CTCF, cohesin, CTCF/cohesion, or Mediator (Fig. 5F), supporting previous findings implicating these factors in long-range interactions (Hadjur et al. 2009; Mishiro et al. 2009; Nativio et al. 2009; Handoko et al. 2011; Phillips-Cremins et al. 2013). The finding that these factors are potentially blocking some interactions while facilitating others suggests that they may contribute to the specificity of promoter contacts.

### Promoter–promoter 3D interaction networks

**Figure 6.**
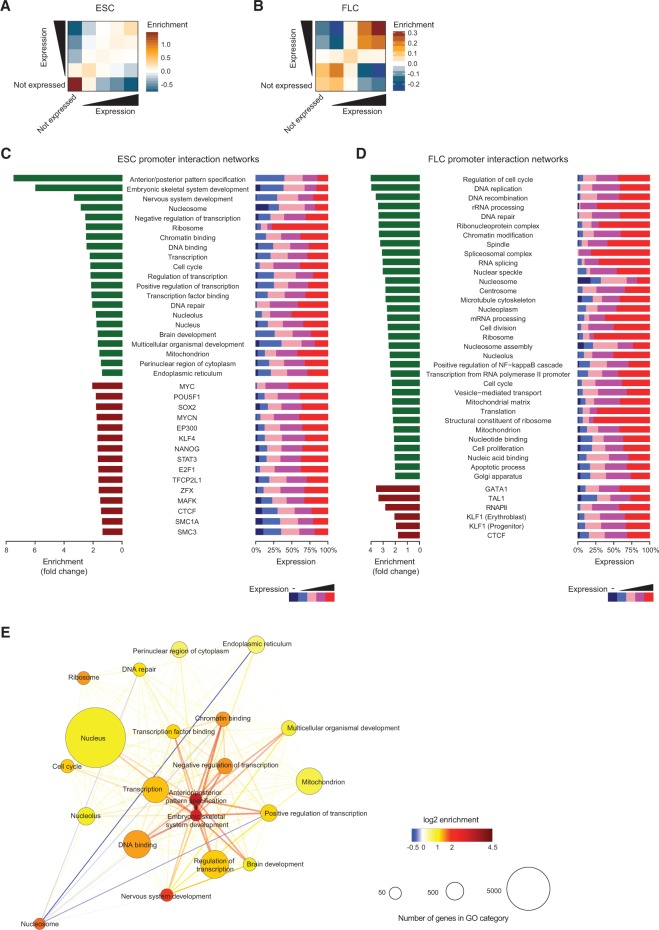
Promoter–promoter interaction networks. (*A*,*B*) Enrichment of interactions between promoters from different expression categories in ESCs (*A*) and FLCs (*B*). (*C*) ESC promoter–promoter interaction networks. (*Left*) Fold enrichment of GO categories (green bars) and promoters bound by *trans-*acting factors (dark red bars) in ESC promoter interaction networks. (*Right*) Distribution of expression categories within the respective ESC promoter interaction networks. (*D*) FLC promoter–promoter interaction networks. (*Left*) Fold enrichment of GO categories (green bars) and promoters bound by *trans-*acting factors (dark red bars) in FLC promoter interaction networks. (*Right*) Distribution of expression categories within the respective FLC promoter interaction networks. (*E*) Connectivity between ESC promoter–promoter subnetworks categorized based on gene ontology. Circle sizes represent the numbers of genes within the respective promoter subnetwork. Color of circles represents the fold enrichment of connectivity between the members, whereas edge colors show the enrichment of connectivity between the subnetworks.

We next used our promoter CHi-C data to interrogate contacts between promoters. Long-range intra- and interchromosomal promoter–promoter contacts may represent an additional layer of 3D genome organization with potential to influence gene expression (Schoenfelder et al. 2010; Fanucchi et al. 2013). Consistent with previous results (Lieberman-Aiden et al. 2009), active and inactive promoters are largely spatially segregated, but surprisingly we found that promoters across the entire expression spectrum preferentially contact other promoters within the same expression category, especially in FLCs (Fig. 6A,B). That highly expressed genes contact each other more often could be predicted by the fact that they are more often at shared transcription sites compared to medium and poorly expressed genes (Schoenfelder et al. 2010). However, the same rationale would not predict that medium and poorly expressed genes would preferentially contact medium and poorly expressed genes, respectively. This nonrandom contact between promoters suggests that the transcriptional output of groups of spatially associating genes may be coordinated.

To characterize these promoter–promoter networks more thoroughly, we interrogated the connectivity of promoters associated with more than 1000 GO terms covering all major cellular functions. To negate potential interaction bias between nearby promoters, we focused the analysis on long-range (>1 Mb) interactions. We found striking differences between ESCs and FLCs, with several subnetworks enriched in specific GO categories and containing promoters with higher than expected connectivity (Fig. 6C,D). The strongest subnetworks present in ESCs (fold change > 6; *P*-value < 8.9 × 10^−17^; colocalization analysis) contain genes related to developmental processes, such as anterior/posterior pattern specification or embryonic development (Fig. 6C). These subnetworks were not found in FLCs, where instead we found subnetworks of genes involved in regulation of cell cycle and DNA replication (Fig. 6D). The majority of genes engaged in promoter–promoter networks in FLCs are expressed at moderate to high levels (Fig. 6D), whereas the spatially associating developmental gene networks in ESCs contain a higher percentage of lowly expressed genes (Fig. 6C). This finding suggests that poised or primed developmental genes spatially associate in the pluripotent genome.

We next analyzed whether promoters occupied by specific transcription factors are engaged in preferential interactions. We found that promoters bound by key transcriptional regulators in the respective cell type (POU5F1, KLF4, SOX2, and NANOG in ESCs; GATA1, KLF1, and TAL1 in FLCs) associate at higher than expected frequencies (Supplemental Table 5). Neither genomic distance nor expression level (Supplemental Fig. 6A,B; Supplemental Table 5) can fully account for the level of promoter connectivity we observe, suggesting that occupancy by specific factors favors associations between specific groups of genes beyond the preferential contacts between expression categories we observed (Fig. 6A,B). In general, promoters occupied by tissue-specific transcription factors show a stronger enrichment in promoter networks than promoters bound by the architectural chromatin proteins CTCF and the cohesin complex (Fig. 6C,D).

Finally, we looked at the degree of contacts between promoter–promoter subnetworks. Interestingly, we found that the MYC, SOX2, POU5F1, and NANOG subnetworks are highly centralized and strongly connected to each other in ESCs (Supplemental Fig. 6C). Visualizing the networks based on GO categories demonstrates that key genes involved in gene expression control and developmental processes are central and highly connected in ESCs, whereas genes in the “nucleosome” and “endoplasmic reticulum” categories have fewer than expected connections (Fig. 6E). The results in FLCs show different categories that are central and highly connected, mainly involved in RNA processing, DNA replication, and repair (Supplemental Fig. 6D). Collectively, these results show that genes involved in related functional pathways, regulated by common transcription factors and of similar transcriptional output are preferentially contacting each other, suggesting that 3D gene organization contributes to coordination of cell type-specific gene expression programs.

## Discussion

### Promoter capture Hi-C: genome-wide promoter interactome profiling

We have applied CHi-C to the ensemble of mouse gene promoters in pluripotent and differentiated cells, providing the first comprehensive catalog linking promoters to their interacting elements across the genomic landscape. CHi-C represents a significant technological advance for the analysis of 3D genome organization. Compared to Hi-C, promoter CHi-C offers a marked increase in resolution for targeted regions, enabling the genome-wide linkage of promoters to their interacting elements with statistical significance. Genome-scale chromosomal interaction maps have previously been generated for selected loci using 4C (Simonis et al. 2006; Zhao et al. 2006). However, even in multiplex 4C experiments (van de Werken et al. 2012; de Wit et al. 2013) the number of interrogated bait points in the genome is considerably smaller (by several orders of magnitude) than in promoter CHi-C. 5C generates high-resolution chromosomal interaction landscape maps of megabase-size genomic regions (Dostie et al. 2006; Nora et al. 2012; Sanyal et al. 2012; Phillips-Cremins et al. 2013), but is not capable of capturing interactions involving DNA sequences outside the 5C target region(s).

ChIA-PET (Fullwood et al. 2009) combines antibody-mediated precipitation with ligation to map chromosomal associations. This depends on the availability and efficiency of suitable affinity reagents and restricts bait choice to genomic regions occupied by a protein of interest. CHi-C on the other hand is relatively unbiased, enabling the comparison of chromosomal interaction profiles for genomic regions regardless of cell type or differences in protein occupancy. Two recent reports use ChIA-PET with an antibody against RNA polymerase II (RNAPII) to map interactions for RNAPII-bound genomic regions, including promoter–enhancer associations (Kieffer-Kwon et al. 2013; Zhang et al. 2013). Both studies show pronounced changes in promoter–enhancer contacts between different cell types, consistent with our findings. However, other findings differ markedly. For example, the strongest ESC promoter–promoter interaction networks between key developmental genes uncovered by CHi-C were not detected by RNAPII ChIA-PET (Kieffer-Kwon et al. 2013; Zhang et al. 2013). These ESC promoter networks contain a high proportion of lowly expressed developmental genes, which is likely to reduce their capture efficiency in RNAPII ChIA-PET. Our data indicate that these interaction networks between lowly expressed developmental genes represent a major feature of genome architecture in pluripotent cells. It remains to be determined whether this spatial genome arrangement facilitates the coordinated transcriptional repression of developmental genes to maintain ESC pluripotency, their coordinated expression during cell lineage commitment, or both.

A recent study reported a multiplex sequence capture approach to enrich 3C libraries for promoter interactions (Capture-C) (Hughes et al. 2014). We found that the percentage of sequence reads representing genuine chromosomal interactions is about 10-fold higher in CHi-C compared to Capture-C, presumably due to the fact that genuine ligation junctions are not pre-enriched in Capture-C. Although the number of promoters we targeted in promoter CHi-C is almost 50 times higher than in Hughes et al. (2014), (22,225 versus 455 promoters), the number of informative sequence reads representing chromosomal interactions per captured promoter is comparable.

Promoter CHi-C serves as proof of principle methodology to obtain high-resolution chromosomal interaction maps for a large number of genomic elements. The design of bait probes for CHi-C can be easily modified for unbiased targeting of other genomic regions, such as enhancers, insulators, or genome-wide binding sites of chromatin proteins.

### Regulatory 3D enhancer–promoter circuitry

Our data highlight the enormous complexity of 3D promoter–enhancer architecture, with promoters often skipping the most proximal enhancer and often interacting with multiple enhancers. These results expand upon previous studies, which have detailed intricate regulatory landscapes at several developmentally regulated genes (Carvajal et al. 2001; Carter et al. 2002; Jeong et al. 2006; Kleinjan et al. 2006; Sagai et al. 2009; Montavon et al. 2011; Marinic et al. 2013), where numerous enhancers with overlapping tissue-specific activities control gene expression. Notably, the experimental deletion of some enhancers results in severe developmental abnormalities (Sagai et al. 2005; Attanasio et al. 2013), whereas in other cases, enhancer deletions have no obvious phenotypic consequences (Ahituv et al. 2007) or lead to only subtle changes of target gene expression levels (Bender et al. 2001; Anguita et al. 2002; Drissen et al. 2010; Ferreira et al. 2013). Integrating 3D promoter–enhancer connectivity data may help to better understand these results.

Our data reveal a positive correlation between the expression level of promoters and the number of interacting enhancers. This finding adds weight to the concept of additive effects of enhancer action and suggests possible models to explain how the activity from multiple enhancers is integrated for gene expression control. For example, do multiple enhancers interact with their target genes simultaneously, creating a more stable complex, or do they interact sequentially, increasing the probability that the gene is in contact with one of the enhancers at any moment in time? Both scenarios may result in prolonging transcriptional “on” cycle of genes (Osborne et al. 2004), by increasing the frequency of transcriptional bursts (Suter et al. 2011), or both. Single-cell approaches (Nagano et al. 2013) may help to distinguish between these possibilities.

Contacts between transcribed genes and enhancers have been shown to occur at specialized subnuclear compartments called transcription factories (Osborne et al. 2004; Schoenfelder et al. 2010). It is therefore conceivable that at least some of the detected promoter contacts are the consequence, rather than the cause, of spatial proximity between active genes and regulatory elements at shared subnuclear compartments.

We found that only a fraction (∼20%) of interactions uncovered by promoter CHi-C are between promoters and annotated enhancers. Like all 3C-based assays, promoter CHi-C detects functional interactions and structural interactions, and we cannot exclude the possibility that some of these interactions are nonfunctional, functionally redundant, or that they confer robustness to gene expression programs in a manner similar to the recently described shadow enhancers (Hong et al. 2008; Frankel et al. 2010). Nonetheless, the high-resolution data generated by promoter capture Hi-C provides a framework to formulate hypotheses and to guide the future experimental dissection of promoter–enhancer circuitry in mammalian genomes, for example by CRISPR-mediated deletion of regulatory regions (Zhou et al. 2014).

### Promoter–promoter 3D interactomes

Our promoter CHi-C data uncovers promoter–promoter networks that are composed of preferential interactions between genes functioning in related biological pathways and bound by the same transcription factors, suggesting that these may be spatial networks of coregulated genes. Several studies have implicated transcription factors in three-dimensional gene clustering. KLF1 has been shown to mediate preferential associations between KLF1-regulated genes in FLCs (Schoenfelder et al. 2010), and a similar role has been reported for KLF4 in ESCs (Wei et al. 2013). Spatial clustering has also been reported between the *Ifnb* gene and NFKB-bound sites upon virus infection (Apostolou and Thanos 2008), between the *Nanog* locus and genes bound by pluripotency factors (Apostolou et al. 2013), for pluripotency factor (NANOG, POU5F1, and SOX2) binding sites in ESCs (de Wit et al. 2013; Denholtz et al. 2013), Polycomb-regulated genes (Denholtz et al. 2013), and for NFKB-regulated genes in response to TNF−alpha stimulation (Papantonis et al. 2010). Notably, experimental removal of a gene from a NFKB-dependent multigene complex was shown to directly affect the transcription of its interacting genes, suggesting that coassociation of coregulated genes may contribute to a hierarchy of gene expression control (Fanucchi et al. 2013). Thus, 3D promoter interaction networks may not only facilitate the coordinated expression control of network members, but also allow for regulatory crosstalk between them.

In summary, in addition to linking genes to their long-range regulatory elements genome-wide, our results on promoter–promoter networks emphasize the potential of genome organization in controlling gene expression. The clustering of coregulated genes at nuclear subcompartments, such as transcription factories or Polycomb bodies, may create nuclear microenvironments that are enriched in specific factors to coordinate the expression or repression of specific groups of genes. How this organization is achieved is a major outstanding question in genome biology.

## Methods

### Tissue isolation and cell culture

J1 (129S4/SvJae) murine ESCs were expanded on irradiated primary embryonic fibroblasts under standard pluripotent conditions (15% FBS) on tissue culture plates coated with 0.1% gelatin. To harvest the cells and remove contaminating feeder cells, ESCs were trypsinized and passaged twice for 30 min each.

Fetal livers were dissected from C57BL/6 mouse embryos at day 14.5 (E14.5) of development. Fetal liver cells were filtered through a cell strainer (70 μm) and directly fixed in formaldehyde.

### Promoter capture Hi-C

Hi-C was performed essentially as described in Belton et al. (2012), with some modifications (see Supplemental Material). To capture Hi-C ligation products containing promoter sequences, 500 ng of Hi-C library DNA was lyophilized using a vacuum concentrator at 45°C and resuspended in 3.4 µL H_2_O. Hybridization blockers (Agilent Technologies) were added to the Hi-C DNA, and hybridization buffer and capture bait RNA were prepared according to the manufacturer's instructions (SureSelect Target Enrichment, Agilent Technologies). In a PCR machine, the Hi-C library DNA/hybridization blockers were heated for 5 min at 95°C, before lowering the temperature to 65°C. Hi-C library DNA was mixed with hybridization buffer (prewarmed for 5 min to 65°C), and subsequently with the custom-designed capture bait system (prewarmed for 3 min to 65°C), consisting of 39,021 biotinylated RNAs targeting the HindIII restriction fragment ends of 22,225 mouse gene promoters (Agilent Technologies, see Supplemental Material for capture bait design). After 24 h at 65°C in the PCR machine, biotin pulldown (MyOne Streptavidin T1 Dynabeads; Life Technologies) and washes were performed following the SureSelect Target enrichment protocol (Agilent Technologies). After the final wash, beads were resuspended in 30 µL NEBuffer 2 without prior DNA elution, and a post-capture PCR (four amplification cycles using Illumina PE PCR 1.0 and PE PCR 2.0 primers) was performed on DNA bound to the beads via biotinylated RNA. Capture Hi-C libraries were paired-end sequenced (HiSeq 1000, Illumina).

### DNA FISH

BAC clones (RP23-162O16 [*Slc25a37* locus], RP23-51D11 [*Dleu2* locus], RP23-369O11 [*Dcaf11* locus], RP23-9O8 [*Tbx3* locus], RP23-438D11 [*Fzd10* locus], RP23-431D16 [*Uncx* locus], RP23-141E23 [*Hist1h4h* locus], RP24-239K5 [*Vmn1r* locus], RP23-73B14 [*Hist1h2ai* locus]) were purchased from Life Technologies or BACPAC Resources (Children's Hospital Oakland). BAC DNA was purified using the NucleoBond BAC100 kit (Macherey-Nagel), and labelled with aminoallyl-dUTP by nick translation. After purification, 0.5–1 µg labeled BAC DNA was coupled with Alexa Fluor 488, Alexa Fluor 555, or Alexa Fluor 647 reactive dyes (Life Technologies) according to the manufacturer's instructions, and DNA FISH was performed as described (Nagano et al. 2013) with minor modifications (see Supplemental Material).

### Interaction calling

Raw sequencing reads were processed using the HiCUP pipeline, which maps the ditags against the mouse genome (mm9), filters experimental artefacts, such as circularized reads and religations, and removes duplicate reads (http://www.bioinformatics.babraham.ac.uk/projects/hicup/). Significantly interacting regions were called using the GOTHiC BioConductor package (http://www.bioconductor.org/packages/release/bioc/html/GOTHiC.html). This assumes that biases occurring in Hi-C-type experiments are captured in the coverage (total number of reads mapping to a genomic region), and significantly interacting regions can be separated from background noise using a cumulative binomial test based on coverage followed by Benjamini-Hochberg multiple testing (FDR < 0.05) (Benjamini and Hochberg 1995). Promoter–promoter and promoter–genome interactions were handled separately. For promoter–promoter interactions, we calculated a modified null distribution to account for the nonmultiplicative capture bias in products targeted by two baits. A random ligation sample (see Supplemental Methods) was used to build a generalized linear model. The product and the sum of the coverage values of the two ends were used as input variables, whereas the interaction frequencies of random ligation events were used as dependent variables. Predicted interaction frequencies for the actual samples were calculated from the model using logit regression. Then we applied the GOTHiC binomial test with this modified background distribution. Significant interactions were further filtered by removing interactions in which one of the fragments has extremely high coverage. We kept interactions for which there is at least one valid ditag with one of the two neighboring fragments to control for spurious interaction spikes. Promoter–genome interactions were considered if they were present in both biological replicates; promoter–promoter interactions were pooled to increase the sensitivity for detecting long-range interactions. Finally, we fitted a normal distribution to the lower peak of the bimodal average log observed/expected distribution and used a cutoff at the 95th percentile (∼10) to remove weak promoter–genome interactions.

## Data access

Raw data and the list of interactions have been submitted to the EBI ArrayExpress (https://www.ebi.ac.uk/arrayexpress/) under accession number E-MTAB-2414.

## Competing interest statement

The authors declare that we have applied for a patent related to the content of this manuscript. The international application number for this patent application is PCT/GB2014/052664.

## Supplementary Material

Supplemental Material
