# Supplementary material for: The pluripotent regulatory circuitry connecting promoters to their long-range interacting elements
Source: Genome Res. 2015 Apr;25(4):582–97. doi: 10.1101/gr.185272.114 (PMC4381529; doi:10.1101/gr.185272.114)
Supplement: Supplemental Material [file supp_25_4_582__index.html]

The pluripotent regulatory circuitry connecting promoters to their long-range interacting elements — Supplemental Material 

# The pluripotent regulatory circuitry connecting promoters to their long-range interacting elements

## Supplemental Material

**Files in this Data Supplement:**

- Supp Material.pdf
- Supp Table 1.xlsx
