# Supplementary material for: The pluripotent regulatory circuitry connecting promoters to their long-range interacting elements
Source: Genome Res. 2015 Apr;25(4):582–97. doi: 10.1101/gr.185272.114 (PMC4381529; doi:10.1101/gr.185272.114)
Supplement: Supplemental Material [file supp_gr.185272.114_SuppMaterial.pdf]

Supplemental Information

Schoenfelder, Furlan-Magaril, Mifsud, Tavares-Cadete, Sugar, Javierre, Nagano, Katsman, Sakthidevi, Wingett, Dimitrova, Dimond, Edelman, Elderkin, Tabbada, Darbo, Andrews, Herman, Higgs, LeProust, Osborne, Mitchell, Luscombe, Fraser

"The pluripotent regulatory circuitry connecting promoters to their long-range interacting elements"

SUPPLEMENTAL TABLES
Table S1: Genomic coordinates of mouse Promoter Capture Hi-C baits
Table S2: Processing of sequence reads
Table S3: Publicly available datasets used
Table S4: Enrichment of chromatin marks in promoter-interacting fragments
Table S5: Promoter-promoter sub-network connectivity

SUPPLEMENTAL METHODS
Hi-C
Bait capture system design for Promoter Capture Hi-C
Quantitative chromosome conformation capture (3C-qPCR)
DNA FISH
Expression analysis
Interaction symmetry
ChIP-seq processing
Enrichment calculation in non-bait interacting fragments
Chromatin states definition
Defining enhancer regions
Enhancer interactions with proximal or distal promoters
Promoter interactions with proximal or distal enhancers
Overlap of enhancer interactions between ESCs and FLCs
Interactions and LADs
TADs and interaction directionality
Promoter co-localization analysis
Promoter interaction networks
Target enrichment controls
Enrichment calculation in bait-bait interactions

REFERENCES FOR SUPPLEMENTAL INFORMATION

SUPPLEMENTAL FIGURES
Figure S1: Promoter CHi-C recapitulates known long-range chromosomal interactions
Figure S2: Validation of Promoter CHi-C by triple label DNA FISH
Figure S3: Characterization of promoter-interacting regions in FLCs
Figure S4: Spatial promoter-enhancer interactions uncovered by Promoter CHi-C
Figure S5: Promoter interactions and chromosomal domains
Figure S6: CHi-C uncovers non-random promoter-promoter interaction networks

SUPPLEMENTAL TABLES

Table S1: Genomic coordinates of mouse Promoter Capture Hi-C baits

Table S2: Processing of sequence reads

Dataset	Total reads	Mappable reads	Mappable captured reads	De-duplicated reads	De-duplicated captured reads
Hi-C ESC	233284330	79532312	5130386	77644932	5010585
Hi-C FLC	206707252	89400314	5142965	88968261	5118072
CHi-C ESC rep1	556132682	143739084	89148208	110845879	59718558
CHi-C ESC rep2	571862439	235577860	180655899	128567174	79907279
CHi-C FLC rep1	316650488	164451580	102362251	155634527	95525187
CHi-C FLC rep2	211932208	108843931	76140872	97232084	65483365
RL CHi-C ESC	142506398	37448163	32786371	28239100	23844267
RL CHi-C FLC	132797921	63915383	51922654	58006229	46418063

Table S3: Publicly available datasets used

Developmental_stage	Tissue/ cell type	Modification/ chromatin protein	Reference
E14.5	liver	CTCF	Shen, et al. Nature (2012)
E14.5	liver	Erythroblast-KLF1	Pilon, et al. Blood (2011)
E14.5	liver	GATA1	Cheng, et al. Genome Research (2009)
E14.5	liver	H3K27ac	Shen, et al. Nature (2012)
E14.5	liver	H3K27me3	Wong, et al. Blood (2011)
E14.5	liver	H3K36me3	Wong, et al. Blood (2011)
E14.5	liver	H3K4me1	Shen, et al. Nature (2012)
E14.5	liver	H3K4me2	Wong, et al. Blood (2011)
E14.5	liver	H3K4me3	Shen, et al. Nature (2012)
E14.5	liver	H3K79me2	Wong, et al. Blood (2011)
E14.5	liver	H3K9ac	Wong, et al. Blood (2011)
E14.5	liver	H3K9me3	ENCODE
E14.5	liver	H4K16ac	Wong, et al. Blood (2011)
E14.5	liver	RNAPII	Shen, et al. Nature (2012)
E14.5	liver	Progenitor-KLF1	Pilon, et al. Blood (2011)
E14.5	liver	TAL1	Kassouf, et al. Genome Research (2010)
mESC	bruce4	CTCF	ENCODE
mESC	bruce4	H3K27ac	Shen, et al. Nature (2012)
mESC	bruce4	H3K27me3	ENCODE
mESC	bruce4	H3K36me3	ENCODE
mESC	bruce4	H3K4me1	Shen, et al. Nature (2012)
mESC	bruce4	H3K4me3	Shen, et al. Nature (2012)
mESC	bruce4	H3K9ac	ENCODE
mESC	bruce4	H3K9me3	ENCODE
mESC	bruce4	RNAPII	Shen, et al. Nature (2012)
mESC	V6.5	MYC	Chen, et al. Cell (2008)
mESC	V6.5	E2F1	Chen, et al. Cell (2008)
mESC	V6.5	ELL3	Lin, et al. Cell (2013)
mESC	V6.5	H3K4me2	Stadler, et al. Nature (2012)
mESC	V6.5	H3K79me2	Young, et al. Young (2008)
mESC	V6.5	KLF4	Chen, et al. Cell (2008)
mESC	V6.5	MED1	Kagey, et al. Nature (2010)
mESC	V6.5	MED12	Kagey, et al. Nature (2010)
mESC	V6.5	NANOG	Chen, et al. Cell (2008)
mESC	V6.5	NIPBL	Kagey, et al. Nature (2010)
mESC	V6.5	MYCN	Chen, et al. Cell (2008)
mESC	V6.5	POU5F1	Chen, et al. Cell (2008)
mESC	V6.5	EP300	Creyghton, et al. PNAS (2010)
mESC	V6.5	SMAD1	Chen, et al. Cell (2008)
mESC	V6.5	SMC1A	Kagey, et al. Nature (2010)
mESC	V6.5	SMC3	Kagey, et al. Nature (2010)
mESC	V6.5	SOX2	Chen, et al. Cell (2008)
mESC	V6.5	STAT3	Chen, et al. Cell (2008)
mESC	V6.5	TFCP2L1	Chen, et al. Cell (2008)
mESC	V6.5	ZFX	Chen, et al. Cell (2008)

Table S4: Enrichment of chromatin marks in promoter-interacting fragments

Chromatin mark	HindIII fragments	ESC interacting fragments
mESC H3K4me1	29289	23089
mESC H3K4me3	15700	12492
mESC RNAPII	3704	3108
mESC H3K27ac	19863	15179
mESC H3K27me3	9763	5962
mESC H3K36me3	50951	40914
mESC H3K9ac	24628	21161
mESC H3K9me3	32384	13769
mESC CTCF	33850	24567
mESC SMC1A	8817	7265
mESC SMC3	9315	7610
mESC H3K4me2	31319	25006
mESC NANOG	13034	7826
mESC POU5F1	4550	3239
mESC SOX2	6322	4311
mESC SMAD1	1726	1097
mESC E2F1	9803	8159
mESC TFCP2L1	21215	15807
mESC ZFX	14668	12100
mESC STAT3	3359	2332
mESC KLF4	13361	10544
mESC MYC	1177	1043
mESC MYCN	4592	3944
mESC NIPBL	437	351
mESC MED1	2284	1961
mESC MED12	3299	2694
mESC ELL3	14560	9973
mESC EP300	27768	21105
mESC H3K79me2	16339	13662
LMR	22034	16100
UMR	18274	14985
DNAse-seq	121187	83944
Non-bait fragments with no mark	591213	181327
Non-bait fragments with one or more marks	209898	135944
Total non-bait fragments	801111	317271

Table S5: Promoter-promoter sub-network connectivity

Factor	sample	connections in network	distance control mean	Fold Change (distance control)	p value (distance control)	expression control mean	Fold Change (expression control)	p value (expression control)
MYC	ESC	11845	5442	2.18	1.34296E-56	7474	1.58	1.11E-35
EP300	ESC	18206	10090	1.80	3.46752E-49	13061	1.39	1.14E-30
MYCN	ESC	45052	24160	1.86	5.8959E-49	31922	1.41	5.54E-51
ZFX	ESC	82051	48020	1.71	8.75558E-48	57585	1.42	5.29E-64
E2F1	ESC	77719	44660	1.74	2.67139E-45	58590	1.33	1.83E-53
KLF4	ESC	60377	33540	1.80	8.45699E-45	38885	1.55	3.25E-59
TFCP2L1	ESC	34067	19650	1.73	1.88427E-42	22783	1.50	1.13E-45
POU5F1	ESC	4166	2171	1.92	4.17581E-39	2849	1.46	1.16E-20
CTCF	ESC	31801	20610	1.54	9.17709E-34	20414	1.56	8.38E-50
NANOG	ESC	3686	2055	1.79	1.03605E-33	2661	1.39	2.85E-14
SOX2	ESC	1293	678.8	1.90	1.97287E-29	842	1.54	2.45E-12
STAT3	ESC	882	497.4	1.77	4.07117E-24	629	1.40	7.37E-09
MAFK	ESC	2582	1615	1.60	2.44518E-23	1901	1.36	2.22E-13
SMC3	ESC	2433	1698	1.43	1.50563E-18	1504	1.62	9.80E-23
SMC1A	ESC	2434	1657	1.47	1.52069E-17	1528	1.59	8.90E-24
GATA1	FLC	1769	493.4	3.59	4.78367E-57	636	2.78	1.26E-42
TAL1	FLC	132	38.92	3.39	9.61791E-24	36	3.66	5.96E-22
KLF1_Erythroblast	FLC	148624	71480	2.08	2.0719E-66	93008	1.60	3.10E-80
KLF1_Progenitor	FLC	142008	73030	1.94	7.4536E-64	95135	1.49	1.06E-67
CTCF	FLC	76501	42820	1.79	1.51743E-56	51881	1.47	1.33E-56

SUPPLEMENTAL METHODS

Hi-C

Cells were fixed in 2% formaldehyde for 10 min, and the reaction was quenched by the addition of ice-cold glycine (0.125 M final concentration). After centrifugation (400xg, 10 min), cells were washed with PBS, spun down (400xg, 10 min), the supernatant was removed, and the cell pellet was flash-frozen in liquid nitrogen and stored at -80˚C. Cell pellets were incubated in 50 ml ice-cold lysis buffer (10 mM Tris-HCl pH 8, 10 mM NaCl, 0.2% Igepal CA-630, protease inhibitor cocktail (Roche)) for 30 min on ice, followed by a centrifugation to pellet cell nuclei (650xg, 5 min). Nuclei were resuspended in 1.25x NEBuffer 2, SDS was added (0.3% final concentration), and nuclei were incubated at 37˚C and 950 rpm (shaking) for one hour. Triton X-100 was added to a final concentration 1.7%, and nuclei were incubated at 37˚C and 950 rpm (shaking) for one hour. After digestion with HindIII (NEB; 1500 units per 5 million cells) overnight at 37˚C and 950rpm (shaking), restriction sites were filled in with Klenow (NEB) using biotin-14-dATP (Life Technologies), dCTP, dGTP and dTTP (all at a final concentration of 30 μM) for 60 min at 37˚C. After addition of SDS (1.42% final concentration) and incubation at 65˚C and 950rpm (shaking) for 25 min, ligation was performed for four hours at 16˚C (50 units T4 DNA ligase (Life Technologies) per 5 million cells starting material) in a total volume of 8.2 ml ligation buffer (50 mM Tris-HCl, 10 mM $MgCl_2$, 1mM ATP, 10 mM DTT, 100 ug/ml BSA, 0.9% Triton X-100). The reversal of crosslinks (65˚C overnight in the presence of Proteinase K), was followed by RNase A treatment and two sequential phenol chloroform extractions of DNA. To generate 'random ligation Hi-C libraries', the ligation step was performed after reversal of crosslinks and DNA purification: 30 to 40 μg of unligated Hi-C DNA was incubated for 12 hours at 16˚C in two ligation reactions, each in a total volume of 200 μl ligation buffer (NEB), in the presence T4 DNA ligase (NEB; 7600 units). After 12 hours, ATP (1 mM final concentration) and T4 DNA ligase (NEB; 4000 units) were added, and the reaction volume was adjusted to 300 μl total with 1 x ligation buffer (NEB). After an additional four hours at 16˚C, the ligase was inactivated (15 min at 65˚C), followed by two sequential phenol chloroform purifications of DNA ligation products.

The concentration DNA was determined using Quant-iT Pico Green (Life Technologies), and 40 µg of Hi-C library DNA were subjected to removal of biotin (T4 DNA polymerase (NEB), 4 hours at 20°C) from non-ligated fragment ends, followed by DNA purification (Qiagen PCR purification kit) and sonication (Covaris E220). Sonicated DNA was end-repaired (T4 DNA polymerase, T4 DNA polynucleotide kinase, Klenow (all NEB)), and a double size selection using AMPure XP beads (ratio SPRI beads solution volume to sample volume to 0.6:1, then 0.9:1) was performed, before dATP-addition with Klenow exo$^-$ (NEB). Biotin-marked ligation products were isolated using MyOne Streptavidin C1 Dynabeads (Life Technologies) in binding buffer (5 mM Tris pH8, 0.5 mM EDTA, 1 M NaCl) for 30 min at room temperature, followed by two washes in binding buffer, and one wash in ligation buffer (NEB). PE adapters (Illumina) were ligated onto Hi-C ligation products bound to streptavidin beads for 2 hours at room temperature (T4 DNA ligase NEB, in ligation buffer, slowly rotating). After washes in wash buffer (5 mM Tris, 0.5 mM EDTA, 1 M NaCl, 0.05% Tween-20) and binding buffer, the DNA-bound beads were resuspended in a final volume of 30 µl NEBuffer 2. Bead-bound Hi-C DNA was amplified with 6 to 8 PCR amplification cycles using PE PCR 1.0 and PE PCR 2.0 primers (Illumina). The concentration and size distribution of Hi-C library DNA after PCR amplification was determined by Bioanalyzer profiles (Agilent Technologies).

Bait capture system design for Promoter Capture Hi-C

A list of transcripts was obtained from the Ensembl BioMart system, detailing the positions and assigned biotype for all transcripts in their gene build. The list was filtered to keep only transcripts with a biotype of protein-coding, non-coding (lincRNA), antisense, snRNA, miRNA or snoRNA. A genomic restriction map was then created to generate a list of all HindIII fragments in the genome, and this was subsequently filtered against the transcript list to keep only restriction fragments which contained the transcriptional start position for one or more transcripts. For each restriction fragment containing a transcription start site, two 120 bp capture probes were designed, one to each end of the fragment. Because of the size selection step following sonication, the probes had to fall entirely within a region no more than 500 bp from the end of the fragment. Each probe was required to have no more than 3 consecutive bases masked by repeatmasker, and they had to have a GC content of 25 to 65% to match the efficient capture range of the SureSelect target enrichment

system (Agilent Technologies). Where multiple probes passed these criteria, the probe nearest the end of the restriction fragment was chosen.

Quantitative chromosome conformation capture (3C-qPCR)

Cells were fixed in 2% formaldehyde for 10 min, and 3C was performed essentially as previously described (Dekker et al. 2002). 3C DNA was purified using an Amicon Ultracel 0.5 ml column. For Promoter Capture Hi-C validation, long-range 3C PCR amplicons were designed by combining a 'bait' primer (located within a Promoter Capture target HindIII fragment) with primers to HindIII fragments uncovered by Promoter Capture Hi-C as either interacting or non-interacting (both Promoter Capture targeted and non-targeted HindIII fragments were tested). To generate a standard curve for 3C-qPCR, the corresponding ligation products were generated from a 3C template library and mixed in equimolar concentrations. In order to control for crosslinking and ligation efficiency within individual 3C libraries, each of the long-range ligation products was normalized against a short-range ligation product derived by PCR using the 'bait' primer in combination with a primer for adjacent HindIII fragment sequences. For both cell types, two biological replicates were analyzed by three technical replicates each. Quantitative PCR was performed using SYBR Green PCR master mix (Applied Biosystems) and a C1000 Thermal Cycler (Biorad). 3C primer sequences are available upon request.

DNA FISH

25 ng of each dye-coupled BAC DNA probe was precipitated with 8 μg of mouse Cot-1 DNA and 10 μg of salmon sperm DNA for each hybridization. Precipitated probes were dissolved in 10 μl of 50% formamide, 10% sodium dextran sulfate, 1xSSC and used for hybridization. Cells were suspended in 50 μl of ice-cold PBS and spotted onto poly-L-lysine slides (Sigma) for 4 min to let the cells settle. The slides were immersed in 4% formaldehyde in PBS for 10 min to fix. The fixation was quenched with 0.1 M Tris-HCl, pH7.4 for 10 min, and the cells were permeabilized with 0.1% saponin, 0.1% Triton X-100 in PBS for 10 min. After washing, the slides were immersed in 20% glycerol in PBS for 20 min and subjected to three cycles of freezing/thawing in liquid nitrogen, followed by washing in PBS. The slides were incubated in 0.1 N HCl for 30 min, washed in PBS, permeabilized again in 0.5% saponin, 0.5% Triton X-100 in PBS for 30 min, washed again, and immersed in 50%

formamide in 2xSSC for 10 min. For hybridization, the probe mixtures (prepared as above) were applied onto the cells, covered with coverslips, heated at 78˚C for 2 min and incubated at 37˚C overnight. After hybridization, the slides were washed in 50% formamide in 2xSSC for 15 min at 45˚C, followed by 0.2xSSC at 63˚C for 15 min, 2xSSC at 45˚C for 5 min, and 2xSSC at room temperature for 5 min. The cells were finally counterstained with DAPI, and coverslips were mounted with Vectashield mounting medium (Vector Laboratories). Images of the DNA FISH signals were captured and analysed with the MetaCyte automated imaging system (MetaSystems). 3D distances between the specified genomic loci were measured for a minimum of 300 alleles per experiment, and used Fisher´s exact and Chi square tests to identify significant differences in the distance distributions.

Expression analysis

Genes/promoters were separated into five expression categories based on the RPKM values from GSM723776 (Shen et al. 2012) and ERR031629 (Ficz et al. 2011) mRNA-seq data for ESC (genes falling into different expression categories in the two data sets were assigned to NA), and GSM661638 mRNA-seq data from Ter119+ cells for FLC (Kowalczyk et al. 2012). RPKM values were obtained using TopHat with default settings (Trapnell et al. 2012). Genes with 0 RPKM formed a separate category (inactive) and all other genes (active) were divided into quartiles according to their RPKM. In analyses where active and inactive genes are compared, we considered 0 RPKM genes to be inactive. Promoter fragments containing multiple Ensembl (Flicek et al. 2014) annotated promoters were either assigned to the appropriate category if all genes on the fragments belonged to the same expression category, or were assigned NA if they differed.

Interaction symmetry

We aligned all promoter fragments which contained a single Ensembl promoter and plotted upstream and downstream interactions, using the midpoint of two interacting fragments as the interaction length. We calculated the proportion of interactions within 5 kb bins, up to a distance of 150 kb from the promoter. We separated promoters according to their expression activity, and compared the spread of interaction distances between active and inactive sets using the Ansari-Bradley test.

ChIP-seq processing

For all ChIP-seq datasets where the raw data were available, the raw reads were mapped to the mouse genome using Bowtie (Langmead et al. 2009), with a seed length of 25 bp, allowing reads that had at most only one mismatched nucleotide in the seed, returning only one possible mapping and with the remaining parameters set to default values. After mapping, MACS (Zhang et al. 2008) was used to call peaks using default parameters. When no raw data were available, the peaks called in the original publication were used.

Enrichment calculation in non-bait interacting fragments

Enrichment for chromatin marks or states and transcription factors in interacting fragments was calculated using the proportion of fragments in a certain group (e.g., fragments that interact with promoters of a certain expression class) that overlap with a peak for the mark, state or transcription factor being analyzed, divided by the proportion of all non-bait fragments that overlap with such a peak. The resulting value was then converted to its log2 value, so that positive values represent an enrichment compared with all non-bait fragments and a negative value represents depletion. An interacting fragment was assigned to an expression class if all the bait fragments it interacts with have been assigned to the same expression class. If it interacts with baits from different classes, it was excluded from the enrichment analyses.

Chromatin states definition

Chromatin states were defined using ChromHMM (Ernst and Kellis, 2012). Data from 30 histone modifications and transcription factors were used to identify eight chromatin states. For downstream analysis, states 2 and 3 were merged and named "Promoter-like" as they showed a high similarity. The enrichment of chromatin states in non-bait interacting fragments was calculated in the same way as the enrichment for individual transcription factors and histone modifications.

Defining enhancer regions

Predicted enhancers active in ESC or in E14.5 fetal liver cells (FLCs) were adopted from Shen et al. 2012. Enhancer positions were defined with a +-1.5 kb range in Shen et al. 2012, therefore we identified enhancer fragments as those overlapping with the 3 kb region of predicted enhancers. Super-enhancer positions were taken from Whyte

et al. 2013, and super-enhancer fragments were defined as those overlapping with these regions.

Enhancer interactions with proximal or distal promoters

We calculated the proportion of enhancer-promoter interactions between the enhancer fragment and the nearest promoter (either upstream or downstream). Interactions in which the given fragment interacted with a distal promoter were separated into two categories: interactions with the adjacent promoter but not the closest one, and interactions skipping other promoter/s.

Promoter interactions with proximal or distal enhancers

We calculated the proportion of promoter-enhancer interactions that were between the promoter fragment and the nearest enhancer (either upstream or downstream). Interactions in which the promoter interacted with a distal enhancer were separated into two categories: interactions with the closest but one enhancer and interactions skipping other enhancer/s. TAD and EPU sequence coordinates were taken from Dixon et al. 2012 and Shen et al. 2012, respectively. The GO analysis was carried out using g:Profiler (Reimand et al. 2011).

Overlap of enhancer interactions between ESCs and FLCs

We calculated the percentage of ESC enhancer interactions per promoter that were present in FLC. For this comparison, only those enhancers that were predicted to be active in both cell types were considered. As controls we calculated the percentage of all ESC promoter-genome interactions per promoter present in FLCs as well as the percentage for a random subset of promoter-genome interactions involving the same number of non-promoter fragments as the shared active enhancer fragments.

Interactions and LADs

Promoter-genome interactions were separated according to where interacting fragments were located with respect to LADs. LADs were defined in Peric-Hupkes et al. 2010. Interactions were grouped into 'Outside LADs' (both promoter and interacting region are located outside LADs), 'Intra-LAD' (both promoter and interacting region located within the same LAD), 'Inter-LAD' (promoter and interacting region located in different LADs), 'Promoter in LAD' (the promoter, but

not the interacting region, located within LAD), and 'Interacting region in LAD' (the interacting region, but not the promoter, located within LAD). A similar classification was done for EPUs.

TADs and interaction directionality

The directionality measure of a bait fragment's interactions was obtained by calculating the proportion of interactions originating from a promoter fragment and contacting a fragment with higher genomic coordinates on the same chromosome, divided by the total number of interactions originating from that promoter fragment. The resulting number was then normalized to a range of -1 to 1, so that promoter fragments with negative directionality ratios have more interactions with fragments placed before it in the genome (according to their genomic coordinates), and a promoter fragment with positive directionality ratios have more interactions with fragments placed after it. Three measures of directionality were calculated for each promoter fragment: one measuring the directionality of all its interactions, another the directionality of its interactions with non-promoter fragments and a third measuring the directionality of its interactions with other promoter fragments.

Promoter co-localization analysis

To measure the enrichment of interactions within a set of promoters, we generated one hundred random promoter sets, with comparable pair-wise distance distribution to the original set. The p-value was acquired by counting the number random control sets that have more interactions than the original set. As interaction counts in control experiments generally follow a near-normal distribution, a T test with 99 degrees of freedom was used to more accurately estimate low p-values. Fold change was derived by dividing the number of interactions in the original set by the average expected number of interactions in the control sets.

Promoter interaction networks

The enrichments of the promoter-promoter interactions between two target groups, namely TF binding or GO terms (Ashburner et al. 2000) x and y were calculated using a measure of how likely a bait fragment is to be part of y in one end, given that the other end is part of x. The resulting proportion is then divided by the likelihood of a bait fragment being part of x at any end of an interaction. Interaction networks were

constructed using these enrichments represented as edge colours. GO terms containing at least 50 genes, and at least 20 interactions in the target cell type were selected for investigation. Most significant GO targets with p-value < 0.01, and fold change > 2 were selected for display. The GO terms in the figures were manually curated to exclude too generic or redundant categories. TFs with significant co-localization (p-value < 0.01) were considered regardless of effect size. The network was laid out using Cytoscape's Edge-Weighted Spring Embedded layout (Shannon et al. 2003).

Target enrichment controls

To further investigate the enrichment of interaction for a given transcription factor, we compared it to a set of control networks of the same size: a) a randomized network with baits in the same expression categories to control for gene expression effects, and b) a randomized network with bait-bait distances comparable to the original set to control for bait proximity. The networks were laid out using Cytoscape's Circle layout (Shannon et al. 2003).

Enrichment calculation in bait-bait interactions

The enrichments among the promoter-promoter interactions were calculated using a measure of how likely an interaction is to have a fragment belonging to expression class y in one end, given that it has a fragment belonging to expression class x in the other end. The resulting proportion is then divided by the likelihood of seeing fragments from expression class x at any end of an interaction and converted to its log2 value.

REFERENCES FOR SUPPLEMENTAL INFORMATION

Ashburner M, Ball CA, Blake JA, Botstein D, Butler H, Cherry JM, Davis AP, Dolinski K, Dwight SS, Eppig JT, et al. 2000. Gene ontology: tool for the unification of biology. The Gene Ontology Consortium. *Nat Genet* **25:** 25-29.

Ernst J, Kellis M. 2012. ChromHMM: automating chromatin-state discovery and characterization. *Nat Methods* **9:** 215-216.

Ficz G, Branco MR, Seisenberger S, Santos F, Krueger F, Hore TA, Marques CJ, Andrews S, Reik W. 2011. Dynamic regulation of 5-hydroxymethylcytosine in mouse ES cells and during differentiation. *Nature* **473:** 398-402.

Flicek P, Amode MR, Barrell D, Beal K, Billis K, Brent S, Carvalho-Silva D, Clapham P, Coates G, Fitzgerald S, et al. 2014. Ensembl 2014. *Nucleic Acids Res* **42:** D749-755.

Langmead B, Trapnell C, Pop M, Salzberg SL. 2009. Ultrafast and memory-efficient alignment of short DNA sequences to the human genome. *Genome Biol* **10:** R25.

Kowalczyk MS, Hughes JR, Garrick D, Lynch MD, Sharpe JA, Sloane-Stanley JA, McGowan SJ, De Gobbi M, Hosseini M, Vernimmen D, et al. 2012. Intragenic enhancers act as alternative promoters. *Mol Cell* **45:** 447-458.

Reimand J, Arak T, Vilo J. 2011. g:Profiler - a web server for functional interpretation of gene lists (2011 update). *Nucleic Acids Res* **39:** W307-315.

Shannon P, Markiel A, Ozier O, Baliga NS, Wang JT, Ramage D, Amin N, Schwikowski B, Ideker T. 2003. Cytoscape: a software environment for integrated models of biomolecular interaction networks. *Genome Res* **13:** 2498-2504.

Trapnell C, Roberts A, Goff L, Pertea G, Kim D, Kelley DR, Pimentel H, Salzberg SL, Rinn JL, Pachter L. 2012. Differential gene and transcript expression analysis of RNA-seq experiments with TopHat and Cufflinks. *Nat Protoc* **7:** 562-578.

Zhang Y, Liu T, Meyer CA, Eeckhoute J, Johnson DS, Bernstein BE, Nusbaum C, Myers RM, Brown M, Li W, et al. 2008. Model-based analysis of ChIP-seq (MACS). *Genome Biol* **9:** R137.

SUPPLEMENTAL FIGURES

Supplemental Fig. 1: Promoter CHi-C recapitulates known long-range chromosomal interactions

(A) Percentage of chromosomal interactions involving promoter elements in raw paired-end sequence reads in Hi-C and Promoter CHi-C data. Hi-C and Promoter CHi-C data were processed using the HiCUP pipeline (Wingett et al. manuscript in preparation). Shown are the percentages of promoter reads in two published Hi-C data sets (Hi-C ESC and Hi-C Cortex; Dixon et al. 2012), and Hi-C (Pre-Capture Hi-C ESC and Pre-Capture Hi-C FLC) and CHi-C data sets (CHi-C ESC and CHi-C FLC) generated in this work.

(B) Absolute number of chromosomal interactions involving promoter elements in raw paired-end sequence reads in Hi-C and Promoter CHi-C data. Hi-C and Promoter CHi-C data were processed using the HiCUP pipeline (Wingett et al. manuscript in preparation). Shown are the numbers of promoter reads in two published Hi-C data sets (Hi-C ESC and Hi-C Cortex; Dixon et al. 2012), and CHi-C data sets (CHi-C ESC and CHi-C FLC) generated in this work. For each data set, promoter reads from two biological replicates were considered.

(C) Promoter CHi-C interaction profile for *Phc1* gene in ESCs. The interaction marked with an arrow has been reported previously in Kagey et al. 2010.

(D) Promoter CHi-C interaction profile for the *Nanog* gene in ESCs. Interactions marked with arrows have been reported previously by Levasseur et al. 2008.

(E) Promoter CHi-C interaction profile for the *Pou5f1* gene in ESCs. The interaction (with a putative enhancer 17 kb upstream of the transcriptional start site of *Pou5f1*) marked with an arrow has been reported previously in van de Werken et al. 2012.

(F) Promoter CHi-C interaction profile for the *Hba* gene in FLCs. Interactions marked with arrows have been reported previously by Vernimmen et al. 2007. The grey rectangle represents an enhancer region deleted by Anguita et al. 2002.

(G) Promoter CHi-C interaction profile for the *Hbb* gene in FLCs. Interactions marked with arrows have been reported previously by Mitchell and Fraser, 2008. Grey rectangles represent regulatory regions deleted by Bender et al. 2001 and Bender et al. 2012.

(H) Promoter CHi-C interaction profile for the *Tal1* gene in FLCs. The grey rectangle represents an enhancer region deleted by Ferreira et al. 2013.

Supplemental Fig. 2: Validation of Promoter CHi-C by triple label DNA FISH

(A) Promoter CHi-C contact maps for a ~13 Mb region on mouse Chromosome 14 in ESCs (upper contact map) and FLCs (lower contact map), encompassing the *Dcaf11*, *Dleu2*, and *Slc25a37* loci. The positions of the gene loci are depicted below the genomic coordinates, and contacts between the regions are marked by blue (contacts between *Slc25a37* and *Dleu2*) and red (contacts between *Slc25a37* and *Dcaf11*) squares respectively, in both CHi-C contact maps.

(B) Representative ESC triple label DNA FISH example, with DNA FISH signals for the *Dcaf11* locus (green), the *Dleu2* locus (purple), and the *Slc25a37* locus (red). Scale bar, 2 μm.

(C) Quantitative analyses of ESC triple label DNA FISH data. The distances between *Slc25a37* and *Dleu2* (represented as blue dots) and between *Slc25a37* and *Dcaf11* (represented as dotted red line) are measured for each allele. Percentages indicate the frequencies of the distance between *Slc25a37* and *Dcaf11* being smaller (above the red dotted line) or greater (below the red dotted line) than the distance between *Slc25a37* and *Dleu2*.

(D) Representative FLC triple label DNA FISH example, with DNA FISH signals for the *Dcaf11* locus (green), the *Dleu2* locus (purple), and the *Slc25a37* locus (red). Scale bar, 2 μm.

(E) Quantitative analyses of FLC triple label DNA FISH data. The distances between *Slc25a37* and *Dleu2* (represented as blue dots) and between *Slc25a37* and *Dcaf11* (represented as dotted red line) are measured for each allele. Percentages indicate the frequencies of the distance between *Slc25a37* and *Dcaf11* being smaller (above the red dotted line) or greater (below the red dotted line) than the distance between *Slc25a37* and *Dleu2*. P-value: Fisher´s exact test to compare the distance distributions between ESCs and FLCs.

(F) Promoter CHi-C contact maps for a ~20 Mb region on mouse Chromosome 5 in ESCs (upper contact map) and FLCs (lower contact map), encompassing the *Uncx*, *Fzd10*, and *Tbx3* loci. The positions of the gene loci are depicted below the genomic coordinates, and contacts between the regions are marked by blue (contacts between *Tbx3* and *Fzd10*) and red (contacts between *Tbx3* and *Uncx*) squares respectively, in both CHi-C contact maps.

(G) Representative ESC triple label DNA FISH example, with DNA FISH signals for the *Uncx* locus (green), the *Fzd10* locus (purple), and the *Tbx3* locus (red). Scale bar, 2 μm.

(H) Quantitative analyses of ESC triple label DNA FISH data. The distances between *Tbx3* and *Fzd10* (represented as blue dots) and between *Tbx3* and *Uncx* (represented as dotted red line) are measured for each allele. Percentages indicate the frequencies of the distance between *Tbx3* and *Uncx* being smaller (above the red dotted line) or greater (below the red dotted line) than the distance between *Tbx3* and *Fzd10*.

(I) Representative FLC triple label DNA FISH example, with DNA FISH signals for the *Uncx* locus (green), the *Fzd10* locus (purple), and the *Tbx3* locus (red). Scale bar, 2 μm.

(J) Quantitative analyses of FLC triple label DNA FISH data. The distances between *Tbx3* and *Fzd10* (represented as blue dots) and between *Tbx3* and *Uncx* (represented as dotted red line) are measured for each allele. Percentages indicate the frequencies of the distance between *Tbx3* and *Uncx* being smaller (above the red dotted line) or greater (below the red dotted line) than the distance between *Tbx3* and *Fzd10*. P-value: Fisher´s exact test to compare the distance distributions between ESCs and FLCs.

Supplemental Fig. 3: Characterization of promoter interactions in FLCs

(A) Composite profile showing the proportion of promoter-genome interactions for 5kb distance bins upstream and downstream of the transcription start sites for active (red) and inactive (blue) promoters in FLCs.

(B) Genomic range of interactions for active (red) and inactive (blue) promoters in FLCs.

(C) Number of promoter-genome interactions in ESCs and FLCs.

(D) Average sequence conservation in the sets of non-bait fragments that interact with promoters in ESCs (green line), in FLCs (blue line) and in all non-bait fragments (red line). Sequence conservation scores were downloaded from the UCSC track of conservation across 30 vertebrate genomes.

(E) Intra- and intergenic distribution of promoter-interacting regions in FLCs, with genes driven by the promoters separated into expression categories. The distribution of intragenic and intergenic sequences in the mouse genome is shown on the right for

comparison.

(F) – (G) Heat maps showing the enrichment/depletion for histone modifications (F), and chromatin proteins (G) in promoter-interacting regions in FLCs, for all promoters separated by expression of the interacting promoters, compared to non-bait regions.

(H) Emission parameters used to define chromatin states in ESCs in this study.

(I) Number of promoters from each expression category interacting with between zero and over ten genomic elements with the hallmarks of enhancers in FLCs.

(J) Example of a promoter (driving the *Klf1* gene) contacting multiple enhancers in FLCs.

(K) Conservation of promoter-genome contacts between ESCs and FLCs. Shown is the percentage of promoters that share 0, 10%, 20%, etc of their interactions with the ensemble of genomic regions. Below: percentage of promoters that share 0, 10%, 20%, etc of their interactions with a random subset of the genomic regions, chosen to match the number of enhancers active in ESCs and FLCs used for the analyses shown in Figure 3K.

Supplemental Fig. 4: Spatial promoter-enhancer interactions uncovered by Promoter CHi-C

(A) Number of interacting promoters for highly connected (HC) enhancers in ESCs and FLCs.

(B) Number and overlap of contacts between promoters and super-enhancers in ESCs, as predicted in Whyte et al. 2013, and by Promoter CHi-C.

(C) Number of interacting promoters for enhancers and super-enhancers in ESCs.

(D) Example of an active gene promoter (*Zmat2*) bypassing enhancers (red arrows) in FLCs.

(E) Pie chart showing the percentages of enhancers interacting with the nearest active promoter (on the linear genomic map), a more distally located promoter, or skipping (at least) one promoter in ESCs and FLCs, as illustrated by schematic on the right (P, promoter; E, enhancer).

Supplemental Fig. 5: Promoter interactions and chromosomal domains

(A) Number of interactions between promoters and enhancer elements with regard to the genomic position of enhancer promoter units (EPUs) in FLCs, separated by expression categories.

(B) Number of promoter-genome interactions with regard to the genomic position of lamina-associated domains (LADs) in ESCs, separated by expression categories.

(C) and (D) Percentage of promoter-genome interactions spanning the boundaries of TADs in ESCs (C) and FLCs (D), separated by expression categories.

(E) Percentage of promoter-genome interactions >500 kb bridging TAD boundaries in ESCs, separated by expression categories. TAD boundary positions are set at 0, and are then shifted artificially in 10 kb steps upstream and downstream.

(F) Number of chromatin protein binding sites in the mouse genome that are bridged (black bars) or not bridged (grey bars), respectively, by any promoter-genome interaction: CTCF binding sites in ESCs (far left), cohesin binding sites in ESCs (left), sites co-occupied by CTCF and cohesin (right), and CTCF binding sites in FLCs (far right).

Supplemental Fig. 6: CHi-C uncovers non-random promoter-promoter interaction networks

(A) NANOG promoter-promoter interaction network in ESCs. Top: Interactions are displayed as lines connecting NANOG-bound promoters displayed on the outer circle of the circos plot. Below left: interactions between a randomized promoter set chosen to match the genomic distances between NANOG promoter interaction network members. Below right: interactions in a randomized promoter set chosen to match the expression levels of NANOG promoter interaction network members. For the distance and expression controls, representative examples are shown. P-values describe differences between the NANOG promoter network and the distance and expression controls, as indicated.

(B) SOX2 promoter-promoter interaction network in ESCs. Top: Interactions are displayed as lines connecting SOX2-bound promoters displayed on the outer circle of the circos plot. Below left: interactions in a randomized promoter set chosen to match the genomic distances between SOX2 promoter interaction network members. Below right: interactions in a randomized promoter set chosen to match the expression levels of SOX2 promoter interaction network members. P-values describe differences between the SOX2 promoter network and the distance and expression controls, as indicated.

(C) Connectivity between promoter sub-networks bound by the indicated trans-acting factors in ESCs. Circle sizes represent the numbers of genes within the respective

promoter sub-network. Colours of circle represents the fold enrichment of connectivity between the members, while edge colours shows the enrichment of connectivity between the sub-networks

(D) Connectivity between FLC promoter-promoter sub-networks categorized based on gene ontology. Circle sizes represent the numbers of genes within the respective promoter sub-network. Colours of circles represent the fold enrichment of connectivity between the members, while edge colours show the enrichment of connectivity between the sub-networks.

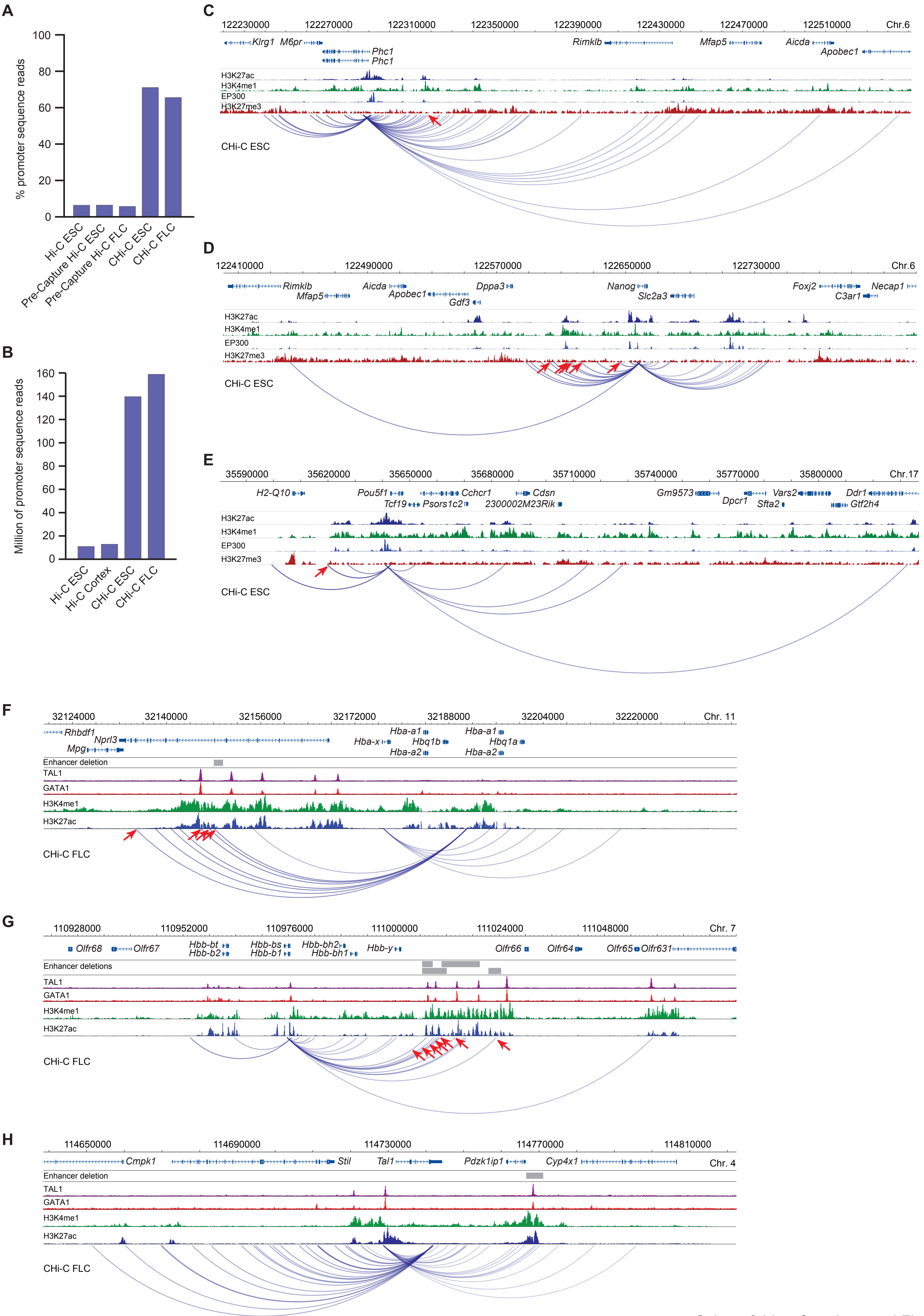

A
% promoter sequence reads
100
80
60
40
20
0
Hi-C ESC
Pre-Capture Hi-C ESC
Pre-Capture Hi-C FLC
CHi-C ESC
CHi-C FLC
B
Million of promoter sequence reads
160
140
120
100
80
60
40
20
0
Hi-C ESC
Hi-C Cortex
CHi-C ESC
CHi-C FLC
C
122230000 122270000 122310000 122350000 122390000 122430000 122470000 122510000 Chr.6
Klrg1 M6pr Phc1 Phc1 Rimklb Mfap5 Aicda Apobec1
H3K27ac
H3K4me1
EP300
H3K27me3
CHi-C ESC
D
122410000 122490000 122570000 122650000 122730000 Chr.6
Rimklb Mfap5 Aicda Apobec1 Dppa3 Gdf3 Nanog Slc2a3 Foxj2 C3ar1 Necap1
H3K27ac
H3K4me1
EP300
H3K27me3
CHi-C ESC
E
35590000 35620000 35650000 35680000 35710000 35740000 35770000 35800000 Chr.17
H2-Q10 Pou5f1 Tcf19 Cchcr1 Psors1c2 Cdsn 2300002M23Rik Gm9573 Dpcr1 Vars2 Sfta2 Ddr1 Gtf2h4
H3K27ac
H3K4me1
EP300
H3K27me3
CHi-C ESC
F
32124000 32140000 32156000 32172000 32188000 32204000 32220000 Chr. 11
Rhbdf1 Nprl3 Mpg Hba-x Hba-a1 Hbq1b Hba-a2 Hba-a1 Hbq1a Hba-a2
Enhancer deletion
TAL1
GATA1
H3K4me1
H3K27ac
CHi-C FLC
G
110928000 110952000 110976000 111000000 111024000 111048000 Chr. 7
Olfr68 Olfr67 Hbb-bt Hbb-b2 Hbb-bs Hbb-b1 Hbb-bh2 Hbb-bh1 Hbb-y Olfr66 Olfr64 Olfr65 Olfr631
Enhancer deletions
TAL1
GATA1
H3K4me1
H3K27ac
CHi-C FLC
H
114650000 114690000 114730000 114770000 114810000
Cmpk1 Stil Tal1 Pdzk1ip1 Cyp4x1 Chr. 4
Enhancer deletion
TAL1
GATA1
H3K4me1
H3K27ac
CHi-C FLC

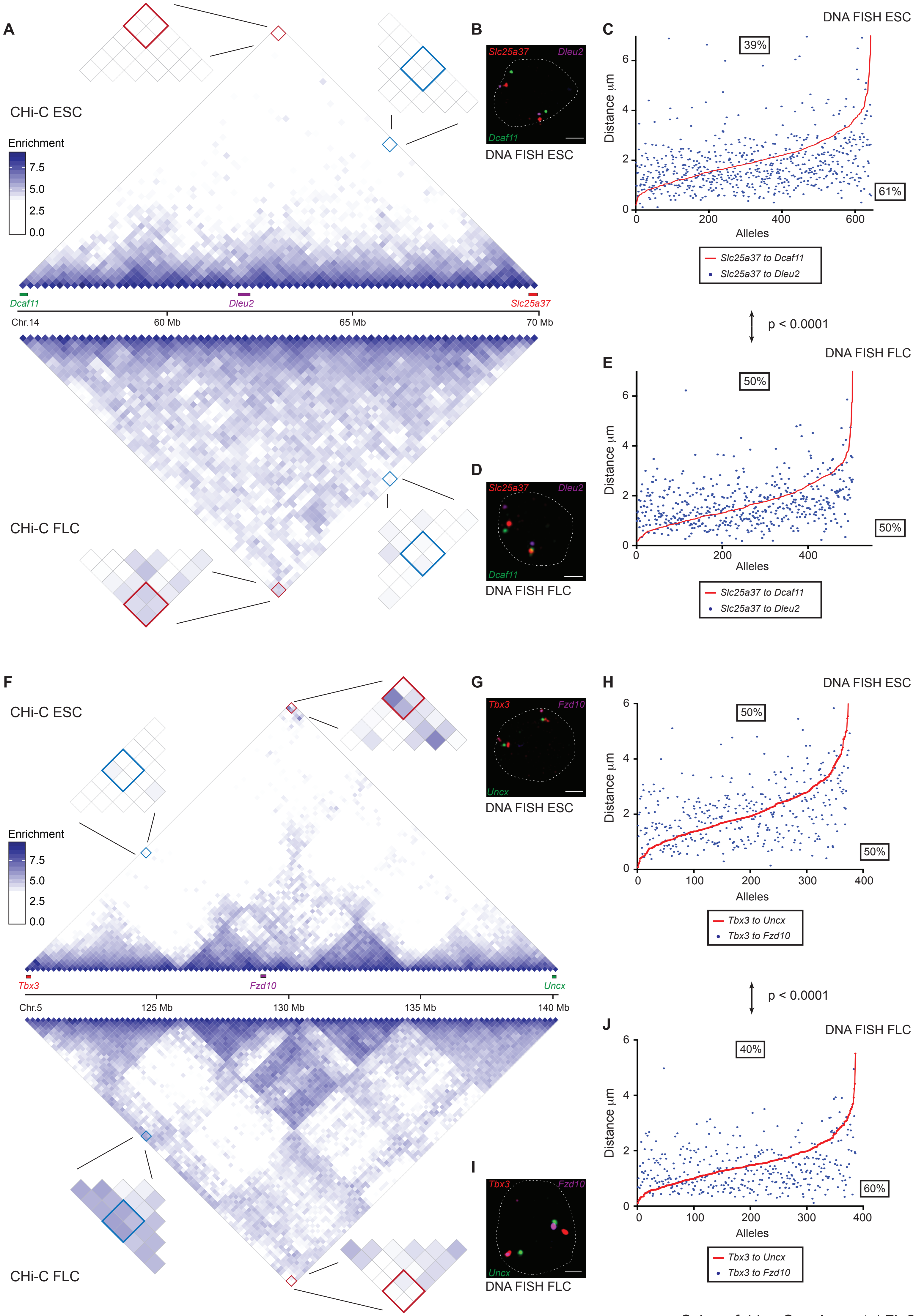

Schoenfelder_Supplemental Fig2

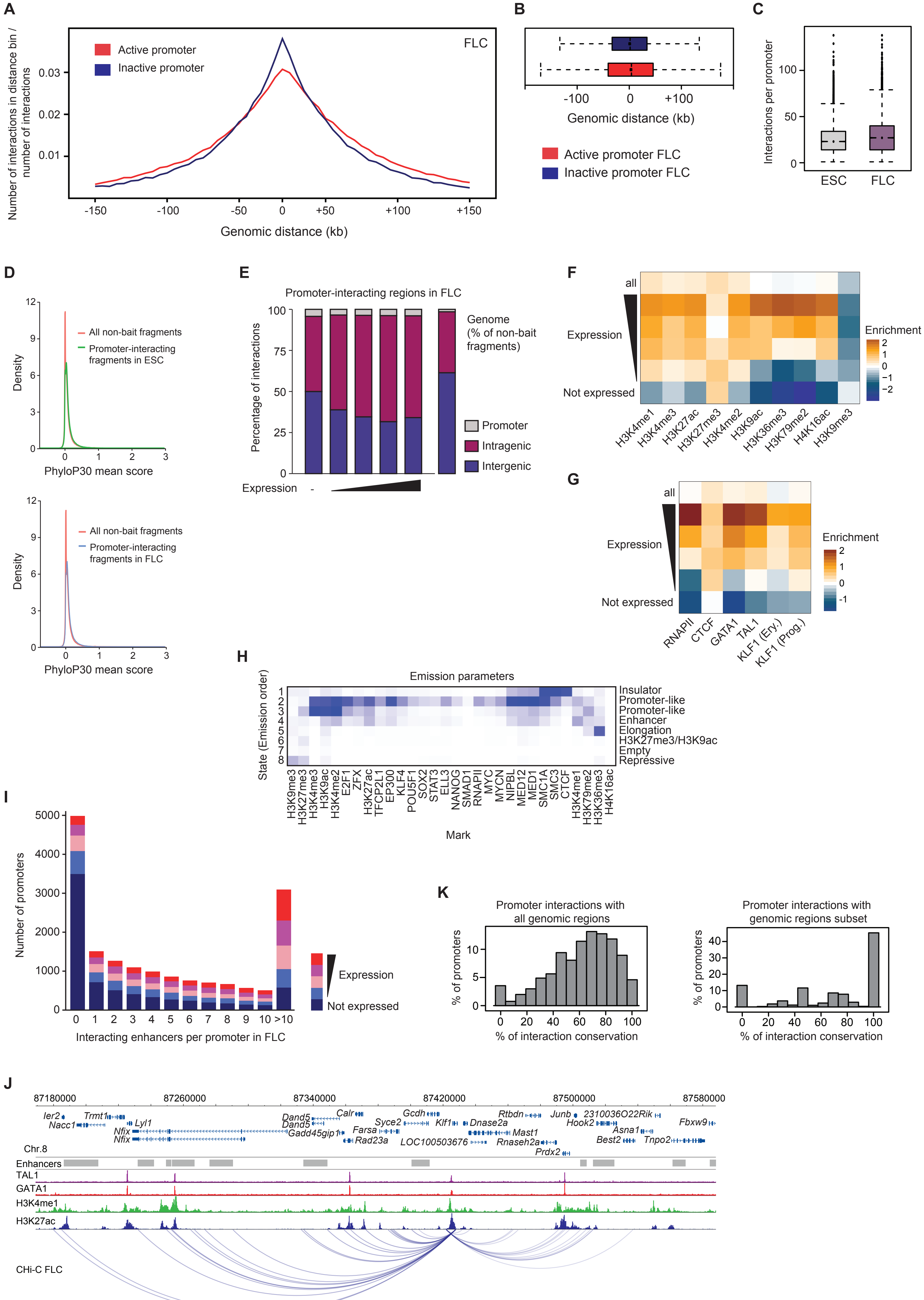

Schoenfelder_Supplemental Fig3

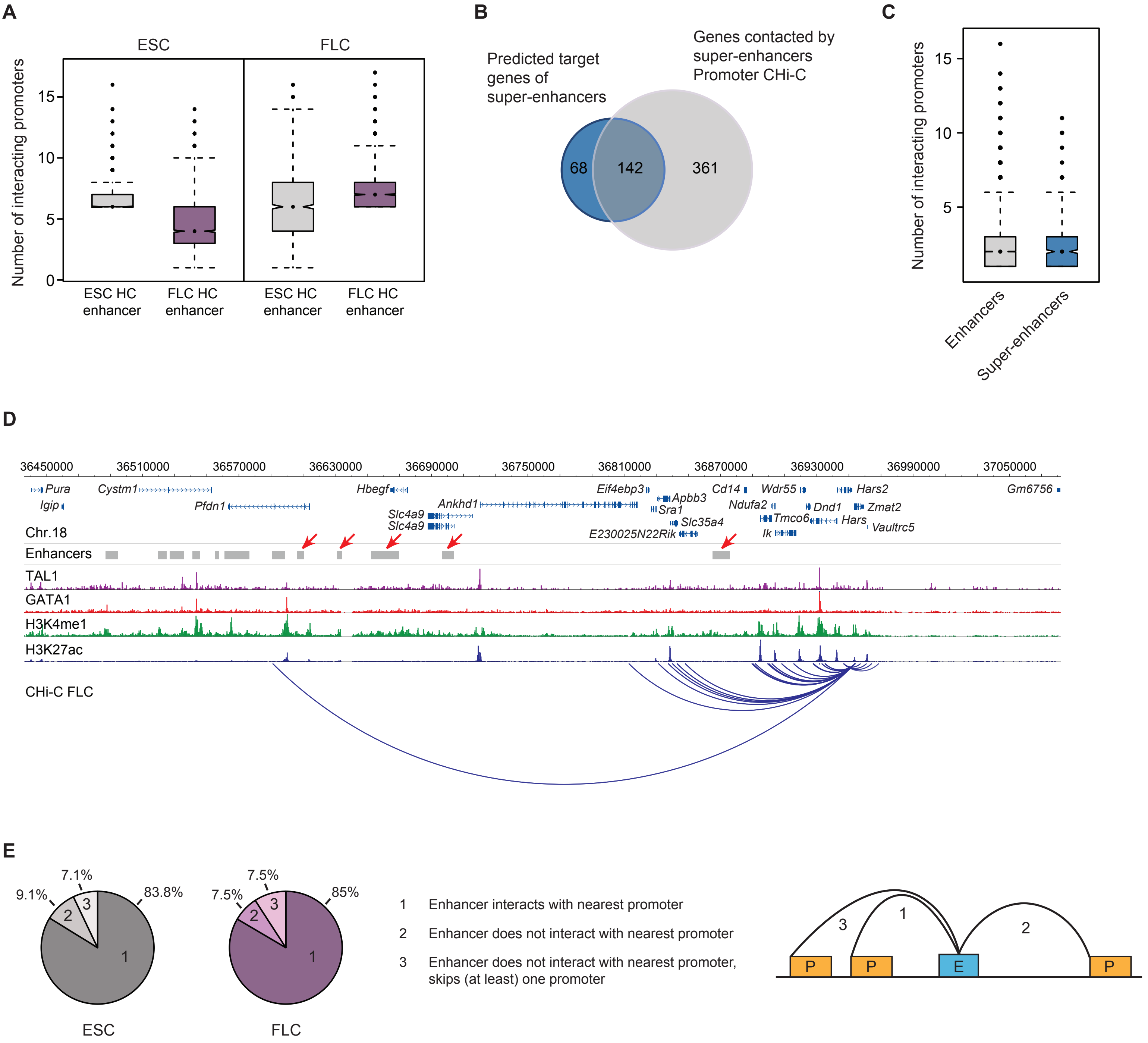

Schoenfelder_Supplemental Fig4

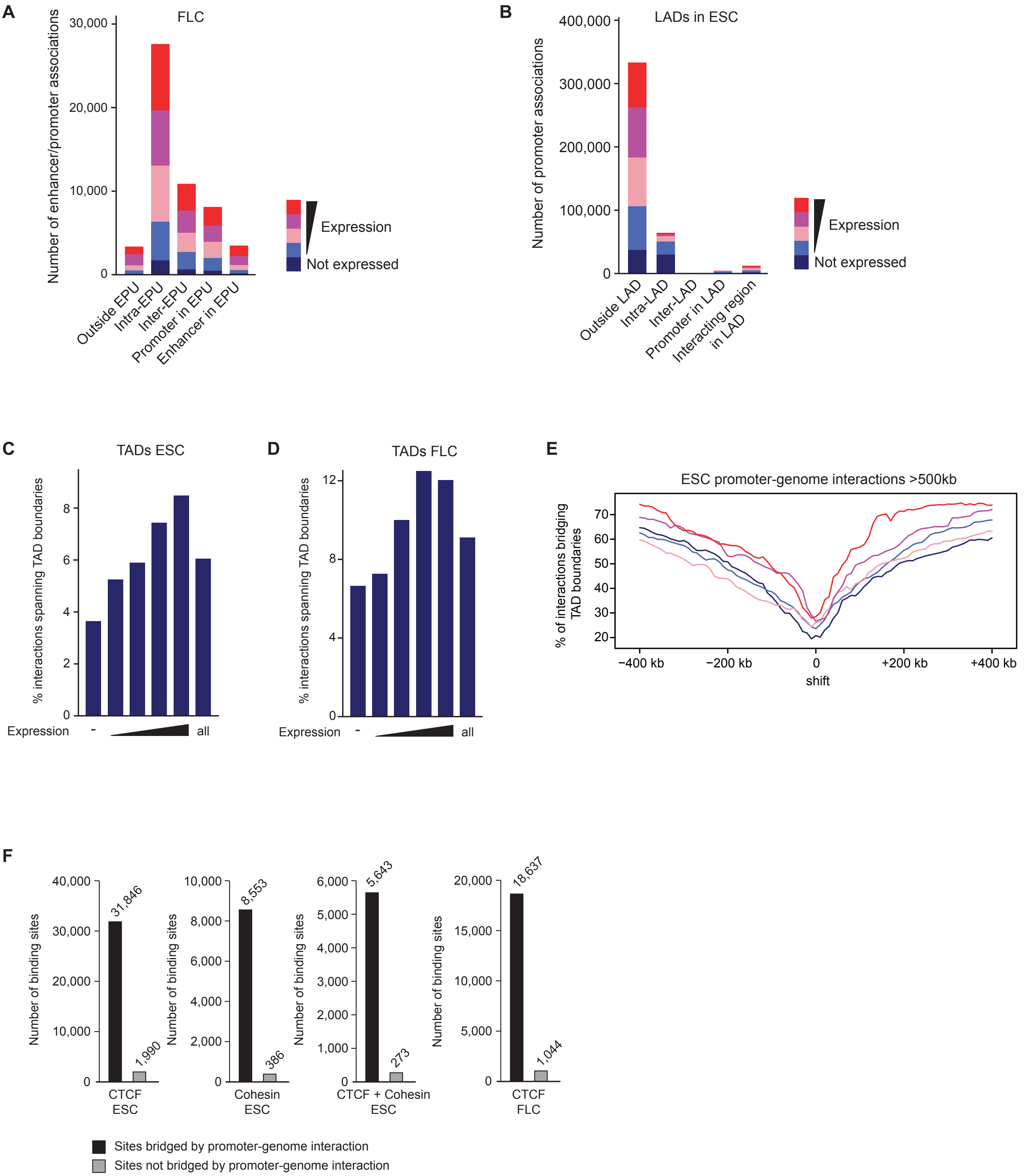
A
FLC
Number of enhancer/promoter associations
30,000
20,000
10,000
0
Expression
Not expressed
Outside EPU
Intra-EPU
Inter-EPU
Promoter in EPU
Enhancer in EPU
B
LADs in ESC
Number of promoter associations
400,000
300,000
200,000
100,000
0
Expression
Not expressed
Outside LAD
Intra-LAD
Inter-LAD
Promoter in LAD
Interacting region in LAD
C
TADs ESC
% interactions spanning TAD boundaries
Expression
-
all
D
TADs FLC
% interactions spanning TAD boundaries
Expression
-
all
E
ESC promoter-genome interactions >500kb
% of interactions bridging TAD boundaries
−400 kb
−200 kb
0
+200 kb
+400 kb
shift
F
Number of binding sites
31,846
1,990
CTCF ESC
8,553
386
Cohesin ESC
5,643
273
CTCF + Cohesin ESC
18,637
1,044
CTCF FLC
Sites bridged by promoter-genome interaction
Sites not bridged by promoter-genome interaction

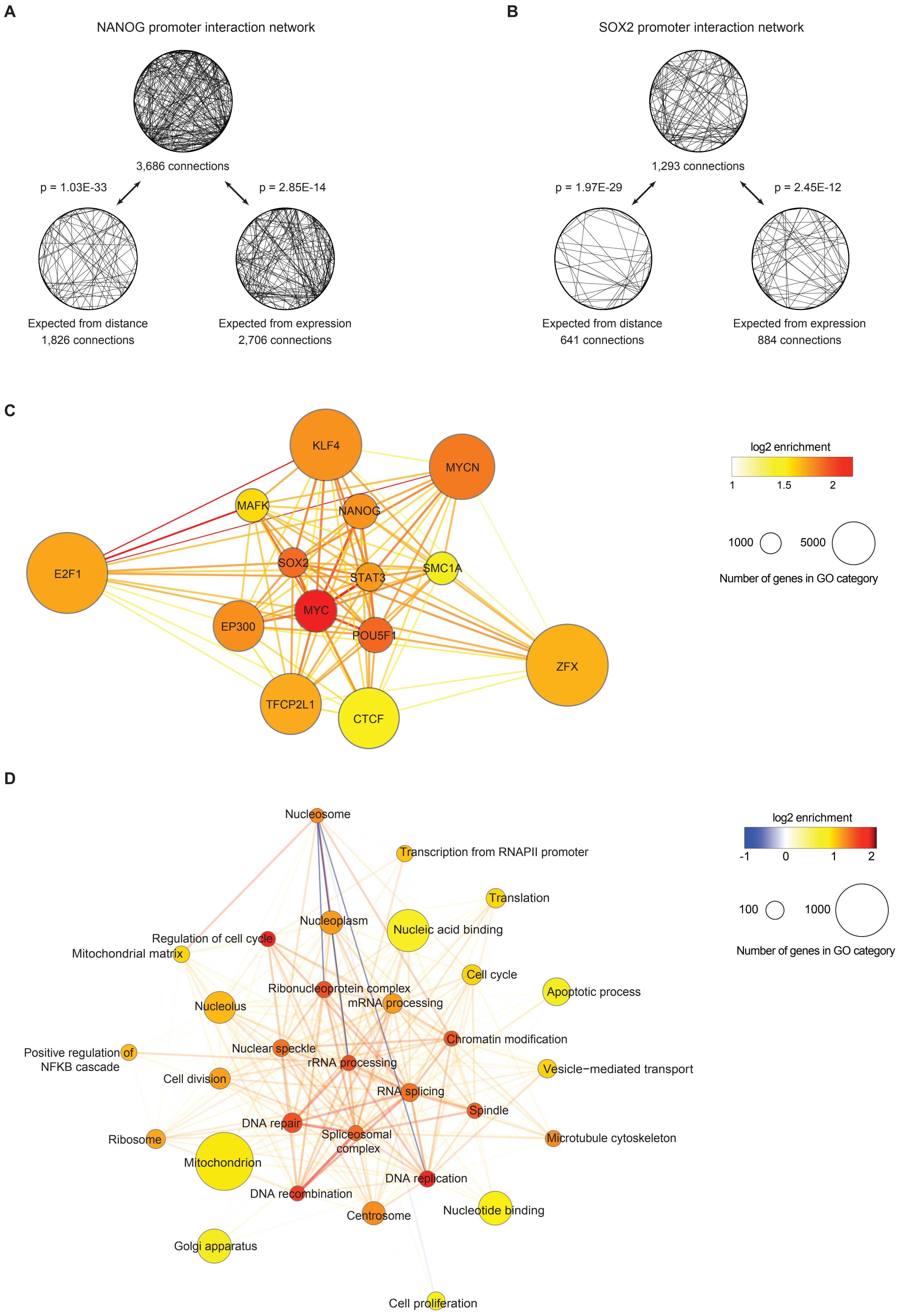

Schoenfelder_Supplemental Fig6
